# Genome-wide identification and characterization of cadmium-responsive microRNAs and their target genes in radish (*Raphanus sativus* L.) roots

**DOI:** 10.1093/jxb/ert240

**Published:** 2013-09-07

**Authors:** Liang Xu, Yan Wang, Lulu Zhai, Yuanyuan Xu, Liangju Wang, Xianwen Zhu, Yiqin Gong, Rugang Yu, Cecilia Limera, Liwang Liu

**Affiliations:** ^1^National Key Laboratory of Crop Genetics and Germplasm Enhancement, College of Horticulture, Nanjing Agricultural University, Nanjing 210095, PR China; ^2^Department of Plant Sciences, North Dakota State University, Fargo, ND 58108, USA

**Keywords:** Cadmium stress, degradome, high-throughput sequencing, microRNAs, Raphanus sativus, transcriptome.

## Abstract

MicroRNAs (miRNAs) are endogenous non-coding small RNAs that play vital regulatory roles in plant growth, development, and environmental stress responses. Cadmium (Cd) is a non-essential heavy metal that is highly toxic to living organisms. To date, a number of conserved and non-conserved miRNAs have been identified to be involved in response to Cd stress in some plant species. However, the miRNA-mediated gene regulatory networks responsive to Cd stress in radish (*Raphanus sativus* L.) remain largely unexplored. To dissect Cd-responsive miRNAs and their targets systematically at the global level, two small RNA libraries were constructed from Cd-treated and Cd-free roots of radish seedlings. Using Solexa sequencing technology, 93 conserved and 16 non-conserved miRNAs (representing 26 miRNA families) and 28 novel miRNAs (representing 22 miRNA families) were identified. In all, 15 known and eight novel miRNA families were significantly differently regulated under Cd stress. The expression patterns of a set of Cd-responsive miRNAs were validated by quantitative real-time PCR. Based on the radish mRNA transcriptome, 18 and 71 targets for novel and known miRNA families, respectively, were identified by the degradome sequencing approach. Furthermore, a few target transcripts including phytochelatin synthase 1 (*PCS1*), iron transporter protein, and ABC transporter protein were involved in plant response to Cd stress. This study represents the first transcriptome-based analysis of miRNAs and their targets responsive to Cd stress in radish roots. These findings could provide valuable information for functional characterization of miRNAs and their targets in regulatory networks responsive to Cd stress in radish.

## Introduction

Cadmium (Cd) is a widespread heavy metal pollutant that is highly toxic to living organisms. Vast areas of agricultural soils are contaminated with Cd via atmospheric deposition and direct application of phosphate fertilizers, animal manure, sewage sludge, and irrigation water (Uraguchi *et al*., 2009; Wiebe *et al*., 2010). Due to its high mobility and water solubility, Cd^2+^ is readily taken up by plant roots and can be translocated into aerial organs. Cd^2+^ inhibits plant growth and development mainly through alterations in photosynthesis, respiration, and nitrogen metabolism, as well as a decrease in water and basic mineral nutrient uptake (Besson-Bard *et al*., 2009). Cd can accumulate in the human body over time via the food chain, resulting in a risk of chronic toxicity to kidney tubules , bone, lungs and some other organs. (Grant *et al*., 2008; Ishikawaa *et al*., 2012). Thus, the elucidation of the regulatory mechanisms underlying Cd uptake, accumulation, translocation, and detoxification is becoming an urgent goal.

MicroRNAs (miRNAs) are a class of endogenous non-coding small RNAs (sRNAs) that regulate gene expression at the transcriptional and post-transcriptional levels by guiding target mRNA cleavage or translational inhibition (Voinnet, 2009). In plants, the primary miRNA transcripts (pri-miRNAs) are translated from nuclear-encoded MIR genes and cleaved by Dicer-like1 (DCL1), leading to the generation of stem–loop miRNA:miRNA* duplexes known as pre-miRNA (Ruiz-Ferrer and Voinnet, 2009). Thereafter, mature miRNAs are bound by the argonaute (AGO) protein to assemble the RNA-induced silencing complex (RISC) (Jones-Rhoades *et al*., 2006). Mature miRNAs guide the RISC to bind to cognate target genes through either cleaving target mRNAs with near perfect complementarity or repressing their translation with lower complementarity (Bartel *et al*., 2004; Brodersen *et al*., 2008). Besides regulating a range of essential cellular and biological processes, a significant fraction of miRNAs have been shown to play crucial roles in plant responses to a variety of abiotic and biotic stresses, such as nutritional deficiency (Pant *et al*., 2009; Liang *et al*., 2010), drought (Zhou *et al*., 2010; Li *et al*., 2011; Wang *et al*., 2011), salinity (Macovei and Tuteja, 2012; Li *et al*., 2013), cold (Zhang *et al*., 2009; Barakat *et al*., 2012), heat (Yu *et al*., 2011; Chen *et al*., 2012*a*), oxidative stress (Sunkar *et al*., 2006), and heavy metal stress (Ding *et al*., 2011; Chen *et al*., 2012*b*; Zhou *et al*., 2012*a*, *b*; Zeng *et al*., 2012).

Target validation is a prerequisite to characterize functionally the biological roles of miRNAs in plants. Modified 5′-rapid amplification of cDNA ends (RACE) has been widely employed for cleavage site mapping and target validation in some species (Jones-Rhoades *et al*., 2006). Nevertheless, this approach is only applicable for target confirmation on a small scale due to it being laborious, low efficiency, and time-consuming (Li *et al*., 2013). Recently, high-throughput degradome sequencing, a method known as parallel analysis of RNA ends (PARE), has been successfully established and adapted to validate miRNA splicing targets in a variety of plant species, such as *Arabidopsis* (Addo-Quaye *et al*., 2008), *Oryza sativa* (Li *et al*., 2010), soybean (Shamimuzzaman *et al*., 2012; Zeng *et al*., 2012), and *Brassica napus* (Zhou *et al*., 2012*a*; M.Y. Xu *et al*., 2012). This technology provides a new efficient strategy to confirm predicted miRNA targets on a large scale in plants (J.H. Yang *et al*., 2013; M.Y. Xu *et al*., 2012).

Understanding heavy metal-regulated gene expression and regulatory networks is a first critical step to elucidate the genetic molecular mechanism of metal accumulation and homeostasis (Khraiwesh *et al*., 2012). Regulation of gene expression by sRNAs at the transcriptional and/or post-transcriptional level is a newly discovered mechanism for plant growth and development and environmental stress responses (Ruiz-Ferrer and Voinnet, 2009; Li *et al*., 2013). Increasing evidence has revealed that miRNA-mediated gene regulation plays a significant role in heavy metal regulatory networks (Ding and Zhu, 2009; Khraiwesh *et al*., 2012; Zhou *et al*., 2012*a*, *b*). Using a direct cloning strategy or real-time PCR (qRT-PCR.)-based analysis, researchers have found that miR171 and miR393 are engaged in response to Cd stress through negatively regulating their targets in *O. sativa* (Huang *et al*., 2009), *Medicago truncatula* (Zhou *et al*., 2008), and *B. napus* (Huang *et al*., 2010). In rice, a total of 19 Cd-regulated miRNAs were identified using miRNA microarray analysis (Ding *et al*., 2011). More recently, high-throughput sequencing technology has become a reliable and efficient tool for functional genomics studies such as genome-wide transcriptome analysis, sRNA sequencing, and gene expression pattern analysis at single-base pair resolution (Morozova and Marra, 2008; McCormick *et al*., 2011). Using this approach, Zhou *et al*. (2012*a*) isolated eight up-regulated and 10 down-regulated miRNA families in response to Cd stress in *B. napus*. Additionally, Al^3+^-mediated miRNAs (four up-regulated and 24 down-regulated) were identified in *M. truncatula* (Chen *et al*., 2012*b*). These findings indicated that a number of miRNAs could play key roles in the regulation of plant responses to heavy metal stress.

Radish (*Raphanus sativus* L., 2*n*=2*x*=18), a major member of the Brassicaceae family, is an important annual or biennial root vegetable crop worldwide (Wang and He, 2005). With a comparative analysis approach, 48 conserved miRNAs belonging to nine miRNA families were isolated from the expressed sequence tag (EST) databases of *R. sativus* (Muvva *et al*., 2012). Moreover, Xu *et al*. (2013) first constructed an sRNA library from radish roots and identified 546 conserved miRNA families as well as 15 novel miRNAs using high-throughput sequencing. Nevertheless, there is no report on systematic identification of Cd-regulated miRNAs and their target genes at the global level in radish. To investigate the roles of miRNAs thoroughly in regulatory networks responsive to Cd stress in radish, two sRNA libraries were constructed from Cd-free and Cd-treated radish roots and sequenced by the Solexa/Illumina system. The aims of this study were to identify Cd-regulated miRNAs from radish roots and to validate the conserved and new target transcripts for Cd-responsive miRNAs by transcriptome-based degradome analysis. The outcomes of this study could enhance our understanding of the miRNA-mediated regulatory networks responsive to Cd stress in radish, and provide new insights into elucidating the molecular genetic mechanisms underlying plant response to Cd stress.

## Materials and methods

### Plant culture and Cd treatment

Seeds of radish advanced inbred line ‘NAU-RG’ were surface sterilized in 1.2% NaOCl and germinated at 25 °C for 3 d in the dark. Germinated seeds were grown in plastic pots and cultured in a growth chamber under 14h light (25 °C)/10h dark (18 °C). After 3 weeks, seedlings were transplanted into a plastic container with modified half-strength Hoagland nutrient solution as previously described (L. Xu *et al*., 2012). The seedlings were treated with 200mg l^–1^ CdCl_2_·2.5H_2_O for 1, 6, 12, 24, and 48h, respectively. Seedlings grown in Cd-free solution were treated as controls. Roots were harvested and immediately frozen in liquid nitrogen and stored at −80 °C. The roots exposed to Cd stress at 12h were further used for Cd-treated sRNA library construction.

### Transcriptome and small RNA sequencing

Total RNA was isolated from Cd-free and Cd-treated roots of radish using Trizol reagent (Invitrogen) according to the manufacturer’s protocols. Equal amounts of RNA from Cd-free and Cd-treated roots (12h) were pooled for transcriptome and degradome library construction. The transcriptome library was prepared using an Illumina TruSeq RNA Sample PrepKit following the manufacturer’s instructions. After removing reads containing only 3′-RNA adaptors and low-quality reads, mRNA transcriptome *de novo* assembly was performed using the SOAP2 program (Li *et al*., 2009). The two sRNA libraries from Cd-free (CK) and Cd-treated (Cd200) roots were prepared based on a previously described procedure (Hafner *et al*., 2008). Briefly, sRNA fragments ranging from 18 to 30 nucleotides (nt) were separated and purified by polyacrylamide gel electrophoresis, and ligated to 5′- and 3′-RNA adaptors by T4 RNA ligase (TaKaRa). The adaptor-ligated sRNAs were subsequently transcribed to single-stranded cDNA using SuperScript II Reverse Transcriptase (Invitrogen). Both sRNA and transcriptome sequencing were performed on a Genome Analyzer II (Illumina, San Diego, CA, USA).

### Analysis of small RNA sequencing data

Clean reads were screened from raw sequencing reads by removing contaminated reads including sequences with 5′-primer contaminants, without the inserted tag, with poly(A) tails, either shorter than 15 nt or longer than 30 nt. The remaining unique RNAs were mapped to the radish reference sequences containing genomic survey sequences (GSS), EST sequences, and the mRNA transcriptome sequences using the SOAP2 program (Li *et al*., 2009). Sequences with a perfect match were retained for further analysis. Sequences matching non-coding RNAs included rRNAs, tRNAs, small nuclear RNAs (snRNAs), and small nucleolar RNAs (snoRNAs) in the Rfam (http://www.sanger.ac.uk/Software/Rfam) and NCBI GenBank (http://www.ncbi.nih.gov/GenBank/) databases (Benson *et al*., 2011) were removed. The remaining unique sequences were aligned with known miRNAs from miRBase 19.0 (http://www.mirbase.org/index.shtml) with a maximum of two mismatches allowed. Mireap software (https://sourceforge.net/projects/mireap/) was used to predict novel miRNAs from the remaining unknown sRNAs. Basic criteria (Meyers *et al*., 2008) were used for screening the potential novel miRNAs. The stem–loop structures of pre-miRNAs were constructed by Mfold (Zuker, 2003).

### Differential expression analysis of miRNAs under Cd stress

The frequency of miRNAs from two libraries was normalized to 1 million by total clean reads of miRNAs in each sample (normalized expression=actual miRNA count/total count of clean reads×1 000 000). If the normalized read count of a given miRNA is zero, the expression value was modified to 0.001 for further analysis. The fold change between the Cd200 and CK library was calculated as: fold change=log_2_ (Cd200/CK). The miRNAs with fold changes >2 or <0.5 and with *P* ≤ 0.05 were considered to be up-regulated or down-regulated in response to Cd stress, respectively. The *P*-value was calculated according to previously established methods (Man *et al*., 2000; Li *et al*., 2011).

### Construction and analysis of degradome libraries

Poly(A) RNA was isolated from 200 µg of total RNA using the Oligotex mRNA mini kit (Qiagen). The degradome library was constructed following a previously described method (German *et al*., 2008, 2009). In brief, polyadenylated transcripts possessing 5′-monophosphates were ligated to an RNA oligonucleotide adaptor containing an *Mme*I recognition site using T4 DNA ligase. Subsequently, first-strand cDNA was generated and amplified with six PCR cycles. Thereafter, the PCR product was digested with *Mme*I and ligated to a 3′ double-stranded DNA adaptor. Finally, the ligated products were amplified with 20 PCR cycles, gel purified, and sequenced on an Illumina Genome Analyzer II.

The raw data were pre-processed to remove adaptor sequences and low-quality sequencing reads, and only 20–21 nt sequences with high quality scores were retained for further subsequent analysis. The degradome reads were mapped to the radish reference sequences as described above. Perfect matching sequences were used to identify potentially sliced miRNA targets by the CleaveLand pipeline (Addo-Quaye *et al*., 2008, 2009). Alignments with no more than five mismatches and no mismatches at the cleavage site (between the 10th and 11th nucleotides) were retained and scored by previously described methods (Allen *et al*., 2005). The identified targets were grouped into three categories based on the relative abundance of the degradome signatures at the miRNA target sites (Addo-Quaye *et al*., 2008).

### qRT-PCR validation

Total RNAs were isolated from six radish samples (0, 1, 6, 12, 24, and 48h) as described above using Trizol (Invitrogen) following the manufacturer’s protocols. Quantitative RT-PCR (qRT-PCR) for miRNAs were performed using the One Step Primer Script^®^ miRNA cDNA Synthesis Kit (Takara) and SuperScript^®^ III Reverse Transcriptase (Invitrogen), respectively. PCRs were carried out in a 20 µl reaction mixture consisting of 2 µl of diluted cDNA, 0.2 µM forward and reverse primer, and 10 µl of 2× SYBR Green PCR Master Mix. The reactions were carried out on an iCycler iQ real-time PCR detection system (BIO-RAD) at 95 °C for 30 s, and 45 cycles of 95 °C for 5 s, 58 °C for 15 s, and 72 °C for 20 s. The threshold cycle (Ct) was determined as the cycle number at which the fluorescence intensity passed a pre-determined threshold. All reactions were assayed in triplicate, and 5.8S rRNA was used as the reference gene. The data were statistically analysed with SAS Version 9.0 software (SAS Institute, Cary, NC, USA) using Duncan’s multiple range test at the *P*<0.05 level of signiﬁcance. The primers for the miRNA qRT–PCR are shown in Supplementary Table S1 available at *JXB* online.

## Results

### Overview of transcriptome and small RNA sequencing

In order to obtain global mRNAs from radish, an mRNA library constructed from total RNAs of radish root was sequenced by the Illumina/Solexa system, resulting in the generation of ~71.95 million raw reads. After the removal of poly(A) tails, short and low-quality tags, and adaptor contamination, 66 110 340 clean reads were obtained. Furthermore, a total of 150 455 contigs with an average length of 299 nt were produced, which were further assembled into 73 084 unigenes with pair-end annotation. These assembled unigenes varied from 200 nt to 8482 nt in length, with an average length of 763 nt. This mRNA transcriptome database, combined with the available GSS and EST sequences released into NCBI databases, make up the radish reference sequences for the prediction of known and novel miRNAs in radish.

To identify miRNAs responsive to Cd in radish, two sRNA libraries were constructed from Cd-free (CK) and Cd-treated (Cd200) roots of radish seedlings, and sequenced by a Solexa/Illumina analyzer. In total, 29.27 million raw reads representing 7.7 million unique sequences were generated in two sRNA libraries (Table 1; Supplementary Fig. S1 at *JXB* online). After removing low-quality tags and adaptor contaminations, 15 779 290 (representing 4 615 663 unique sequences) and 13 495 250 (representing 4 071 113 unique sequences) clean reads ranging from 15 nt to 30 nt were obtained for CK and Cd200 libraries, respectively (Table 2; Supplementary Table S2). A total of 720 362 (CK) and 595 759 (Cd200) unique sequences were successfully mapped to the radish reference sequences, respectively. Thereafter, the non-coding RNAs, including rRNAs, tRNAs, snRNAs, and snoRNAs, were annotated and removed. Querying the remaining sequences against miRbase 19.0 identified 31 280 (CK) and 28 788 (Cd200) unique reads matchings known miRNAs. There remained 4 446 875 (CK) and 3 862 789 (Cd200) unannotated unique sRNA sequences to be screened for identification of novel miRNAs (Table 3).

**Table 1.** Summary of common and speciﬁc sequences between CK and Cd200 sRNA libraries

Class	Unique sRNAs	Percentage	Total sRNAs	Percentage
Total_sRNAs	7 703 916	100.00%	29 274 540	100.00%
CK&Cd200	982 860	12.76%	20 458 468	69.88%
CK_speciﬁc	3 632 803	47.16%	5 042 275	17.22%
Cd200_speciﬁc	3 088 253	40.09%	3 773 797	12.89%

**Table 2.** Statistical analysis of sequencing reads from the CK and Cd200 sRNA libraries in radish

	Total sRNAs	Unique sRNAs
CK		
Raw reads	15 983 864	
Clean reads	15 779 290	4 615 663
Mapped to genomic	7 396 733	720 362
Match known miRNAs	1 771 311	31 280
Unannotated sRNAs	10 536 202	4 446 875
Cd200		
Raw reads	13 664 651	
Clean reads	13 495 250	4 071 113
Mapped to genomic	6 592 663	595 759
Match known miRNAs	1 152 100	28 788
Unannotated sRNAs	8 038 038	3 862 789

Libraries for CK and Cd200 were constructed from *R. sativus* roots exposed to solution without Cd and containing 200mg l^–1^ Cd (pH 5.5), respectively.

**Table 3.** Distribution of small RNAs among different categories in radish

Category	CK		Cd200	
	Unique sRNAs	Total sRNAs	Unique sRNAs	Total sRNAs
Total small RNAs	4 615 663 (100%)	15 779 290 (100%)	4 071 113 (100%)	13 495 250 (100%)
miRNA	31 280 (0.68%)	1 771 311 (11.23%)	28 788 (0.71%)	1 152 100 (8.54%)
rRNA	118 421 (2.57%)	3 186 388 (20.19%)	155 413 (3.78%)	3 328 122 (24.66%)
snRNA	6539 (0.14%)	39 651 (0.25%)	5233 (0.13%)	21 793 (0.16%)
snoRNA	2354 (0.05%)	5761 (0.04%)	1796 (0.04%)	4020 (0.03%)
tRNA	10 194 (0.22%)	239 977 (1.52%)	17 094 (0.42%)	951 177 (7.05%)
Unannotated	4 446 875 (96.34%)	10 536 202 (66.77%)	3 862 789 (94.88%)	8 038 038 (59.56%)

**Fig. 1.**
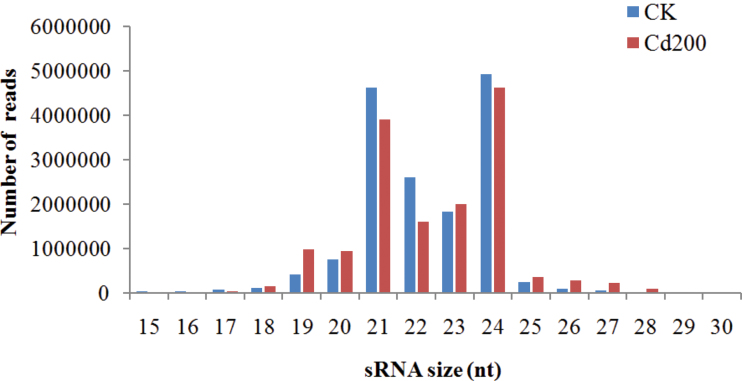
Size distribution of small RNAs in Cd-free (CK) and Cd-treated (Cd200) libraries from radish roots. (This figure is available in colour at *JXB* online.)

The majority of total sRNA reads ranged from 20 nt to 24 nt in length, and 21 nt and 24 nt sequences were dominant in both libraries (Fig. 1). The 24 nt sRNAs were the most abundant, making up 31.53% (CK) and 29.59% (Cd200) of the total sequence reads. This result was consistent with that previously reported for other plant species, such as *Arabidopsis* (Hsieh *et al*., 2009), *Oryza* (Jeong *et al*., 2011), *Medicago* (Lelandais-Brière *et al*., 2009; Wang *et al*., 2011), and *Populus* (Chen *et al*., 2012*a*). Moreover, the fractions of these tags were significantly different between the two libraries. The relative abundances of both 21 nt and 22 nt sRNAs in the Cd200 library were markedly lower than those in the CK library, suggesting that both of the miRNA classes might be repressed under Cd stress. Nevertheless, the abundance of 23 nt sRNAs was relatively larger in the Cd200 compared with the CK library, indicating that the 23 nt sRNAs may play more important roles in plant response to Cd stress.

### Identification of known miRNA families in radish

**Fig. 2.**
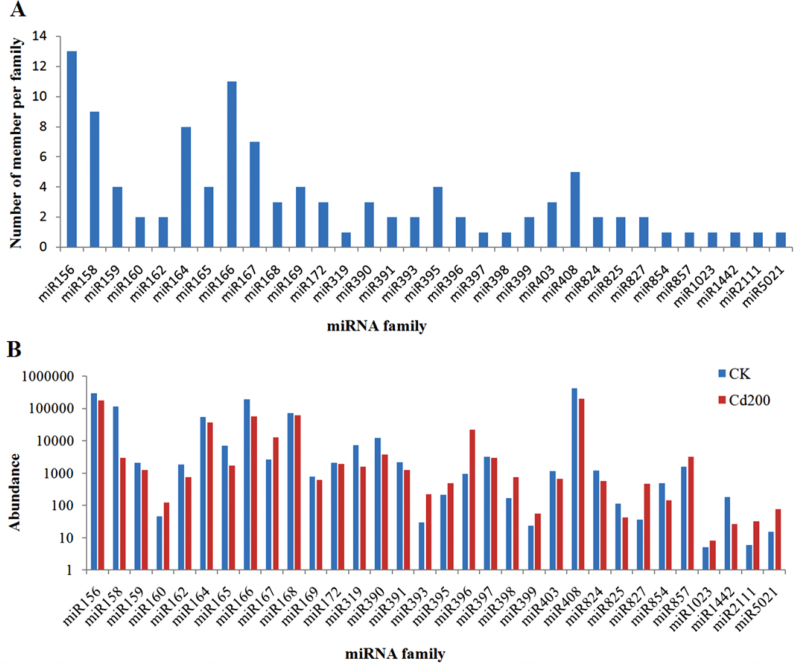
Sizes and abundance of identified known miRNA families from radish. (A) Distribution of known miRNA family size in radish. (B) Counts of each known miRNA family in radish. (This figure is available in colour at *JXB* online.)

Based on mapping the unique sRNA sequences to miRBase 19.0 with a maximum of two mismatches, 42 308 and 38 313 known miRNA sequences were identified in the CK and Cd200 libraries, respectively (Table 2). A total of 93 unique sequences belonging to 16 conserved miRNA families were identified in the two libraries (Table 4; Supplementary Table S3 at *JXB* online). Among these conserved miRNA families, miR156, miR166, miR158, and miR164 contained 13, 11, nine, and eight members, respectively; whereas three miRNA families including miR319, miR397, and miR398 had only one member. Moreover, 15 unique sequences belonging to 10 non-conserved miRNA families were also detected from both libraries. The majority of these non-conserved miRNA families comprised only one member, whereas three miRNA families (miR824, miR825, and miR827) and miR403 contained two and three members, respectively (Fig. 2A).

**Table 4.** Known miRNA families and their transcript abundance identified from CK and Cd200 libraries in radish

Family	No. of members	miRNA reads		Total miRNA reads	Ratio (Cd200/CK)
		CK	Cd200		
Conserved miRNA					
miR156	13	291 882	175 380	467 262	0.60
miR158	9	114 972	2904	178 876	0.03
miR159	4	2081	1233	3314	0.59
miR160	2	45	122	167	2.71
miR162	2	1861	742	2603	0.40
miR164	8	54 932	36 291	91 223	0.66
miR165	4	6986	1707	8693	0.24
miR166	11	189 669	57 169	246 838	0.30
miR167	7	2628	12 794	15 422	4.87
miR168	3	72 829	61 084	133 913	0.84
miR169	4	783	604	1387	0.77
miR172	3	2103	1933	4036	0.92
miR319	1	7241	1572	8813	0.22
miR390	3	11 993	3748	15 741	0.31
miR391	2	2157	1228	3385	0.57
miR393	2	30	224	254	7.47
miR395	4	210	477	687	2.27
miR396	2	950	21 753	5773	22.90
miR397	1	3252	3013	6265	0.93
miR398	1	164	753	917	4.59
miR399	2	23	55	78	2.39
miR408	5	417 843	201 889	619 732	0.48
Non-conserved miRNA					
miR403	3	1168	659	1 827	0.56
miR824	2	1178	568	1746	0.48
miR825	2	115	42	157	0.37
miR827	2	36	472	508	13.11
miR854	1	485	145	630	0.30
miR857	1	1598	3162	5760	1.98
miR1023	1	5	8	13	1.6
miR1442	1	183	26	209	0.14
miR2111	1	6	32	38	5.33
miR5021	1	15	76	91	5.07

The read number differed drastically among the 26 known miRNA families. A few conserved miRNA families, such as miR156, miR166, miR168, and miR408, showed extraordinarily high expression levels in both libraries. miR408 was the most abundant, with 417 843 (CK) and 201 889 (Cd200) reads accounting for 35.3% and 34.1% of all conserved miRNA reads, respectively (Fig. 2B); miR166 was the second most abundant in both libraries. Several miRNA families including miR164, miR172, miR319, miR390, and miR397 had moderate abundance. Nevertheless, a few non-conserved miRNA families, such as miR827, miR1442, miR2111, and miR5021, showed relatively lower expression levels, which were represented by <100 reads in the CK or Cd200 library (Supplementary Table S3 at *JXB* online). Moreover, different members in the same miRNA family also displayed significantly different expression levels. For instance, the abundance of miR156 members varied from 10 to 197 470 reads. These results indicated that the expression level of conserved and non-conserved miRNAs varies dramatically in radish, which was in agreement with previous studies reporting that the non-conserved miRNAs were represented by relatively lower levels than conserved miRNAs (Jeong *et al*., 2011; Yu *et al*., 2012; J.H. Yang *et al*., 2013).

### Identification of novel candidate miRNAs in radish

Based on the criteria for the annotation of novel miRNAs (Meyers *et al*., 2008), a characteristic stem–loop precursor is a prerequisite for the annotation of a new miRNA. In total, 28 novel miRNAs representing 22 unique miRNA sequences were identified with complementary miRNA* as potentially novel miRNAs. The majority of these novel miRNAs were produced from one locus, whereas rsa-miRn5, rsa-miRn9, and rsa-miRn14 were generated from two, three, and four loci, respectively (Table 5). The length of the 22 novel unique miRNAs ranged from 21 nt to 23 nt, with the majority being 21 nt long (17; 77.3%). The length of the novel miRNA precursors varied from 72 nt to 208 nt, with an average of 134 nt. The average minimum free energy (MFE) value was –46.5 kcal mol^–1^, with a range of –86.7 kcal mol^–1^ to –18.2 kcal mol^–1^. The secondary structures of the 22 novel miRNA precursors are shown in Supplementary Fig. S2 at *JXB* online. The expression levels of novel miRNAs and their miRNA* varied significantly between the CK and Cd200 library (Table 5; Supplementary Table S4 at *JXB* online), demonstrating the complexity of miRNA generation and expression under Cd stress in radish.

**Table 5.** Novel miRNAs and their transcript abundance identiﬁed from CK and Cd200 sRNA libraries in radish

miRNA	Mature sequence (5′–3′)	Size	LP	MFE	miRNA reads		Log_2_ (Cd200/CK)	Total miRNA reads	Total miRNA* reads	Loci
					CK	Cd200				
rsa-miRn1	UCGCUUGGUGCAGGUCGGGAA	21	141	–72.20	8263	9468	0.42	17731	199	1
rsa-miRn2	UGAAGCUGCCAGCAUGAUCUA	21	118	–48.00	1524	4358	1.74	5882	16	1
rsa-miRn3	AAGCUAGAGACUUAAAACAAG	21	139	–23.95	65	8	–2.80	73	19	1
rsa-miRn4	GCGUAUGAGGAGCCAAGCAUA	21	106	–49.30	1045	574	–0.64	1619	168	1
rsa-miRn5	GUGGUGACGGUGGUGGUGCGA	21	99	–36.40	5	0	–8.31	5	1	2
rsa-miRn6	CAGGGAACAAGCAGAGCAUGG	21	110	–46.10	3972	2741	–0.31	6713	1557	1
rsa-miRn7	AUAUACUGAAGUUUAUACUCU	21	208	–37.00	58	362	2.87	420	36	1
rsa-miRn8	GUAUGAGGAGCCAAGCAUAU	21	107	–46.60	1137	563	–0.79	1700	198	1
rsa-miRn9	GUACGACGAAGAUGAGCCGACA	23	110	–19.60	36	25	–0.30	61	5	3
rsa-miRn10	UGGAGGCAGCGGUUCAUCGAUC	22	140	–45.70	378	452	0.48	830	2370	1
rsa-miRn11	GCUCAAGAAAGCUGUGGGAAA	21	147	–39.04	0	242	14.18	242	72	1
rsa-miRn12	AAACUGCCUAAACAAACAUAUC	22	171	–40.44	56	38	–11.87	59	14	1
rsa-miRn13	GCUGGAGGCAGCGGUUCAUCGAUC	23	142	–46.80	827	2326	1.72	3153	300	1
rsa-miRn14	AGAUGACAGUGAGGCUUCUUA	21	108	–18.20	0	28	11.02	28	1	4
rsa-miRn15	CCCGCCUUGCAUCAACUGAAU	21	137	–66.30	222	34	–2.48	256	19	1
rsa-miRn16	UCGCUUGGUGCAGGUCGGGAC	21	142	–73.60	12154	14602	1.31	37886	3276	1
rsa-miRn17	UUGGACUGAAGGGAGCUCCUU	21	201	–86.70	8	0	–8.99	8	1	1
rsa-miRn18	CGCCUUGCAUCAACUGAAUCA	21	108	–56.70	1721	1423	1.71	6544	170	1
rsa-miRn19	AUGGAUGUAUGAUAUGAUGGA	21	136	–41.40	0	21	10.60	21	3	1
rsa-miRn20	GGAAUGUUGUUUGGCUCGAAG	21	72	–20.00	10	27	1.66	37	1	1
rsa-miRn21	UCGGACCAGGCUUCAUUCCCC	21	133	–67.20	36853	53357	0.76	90210	436	1
rsa-miRn22	AAGCUGCCAGCGUGAUCUUAAC	22	101	–41.80	2638	3280	0.54	5918	2018	1

LP (nt), The length of precursor; MFE (kcal mol^–1^), minimal folding free energy.

### Identification of Cd-responsive miRNAs in radish

**Fig. 3.**
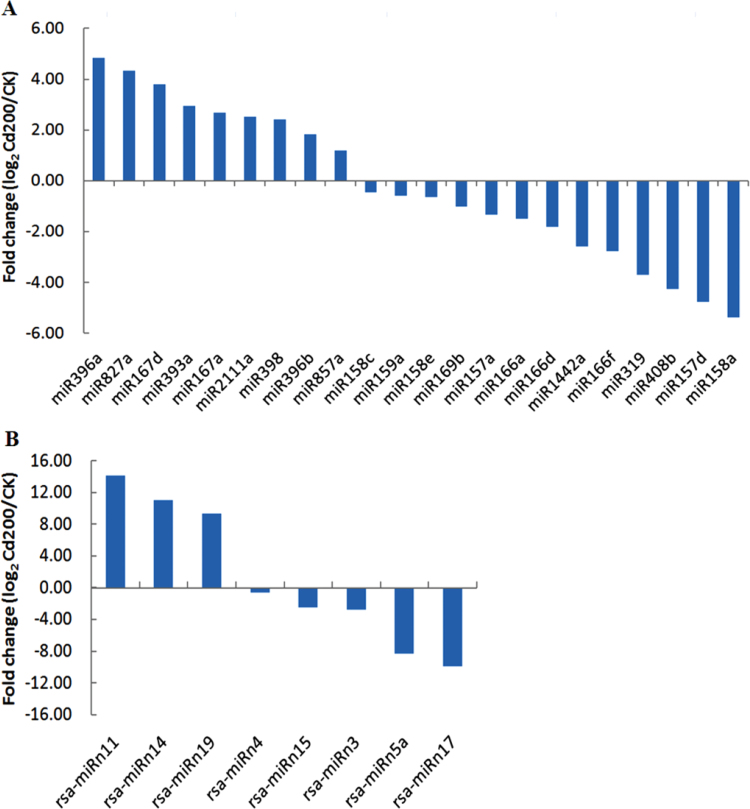
Validation and comparative relative expression of differentially expressed known (A) and novel (B) miRNAs between the CK and Cd200 libraries in radish by qRT-PCR. (This figure is available in colour at *JXB* online.)

To identify differently regulated miRNAs under Cd stress in radish, a differential expression analysis of miRNAs between the CK and Cd200 library was performed. A total of 22 known miRNAs belonging to 15 miRNA families and 11 novel miRNAs belonging to eight miRNA families were identified to be differentially expressed in response to Cd stress (Supplementary Table S5 at *JXB* online). The majority of these Cd-responsive miRNAs were down-regulated in the Cd200 library compared with the CK library, whereas five known miRNAs (miR167a, miR167d, miR396a, miR396b, and miR398) and three novel miRNAs (rsa-miRn3, rsa-miRn11, and rsa-miRn19) showed up-regulated patterns (Fig. 3A, B), suggesting that the down-regulation of miRNAs might play more important roles in plant responses to Cd stress (Khraiwesh *et al*., 2012). The miRNAs with the greatest change in expression levels were rsa-miRn11 and rsa-miRn19, with ratios of 14.18-fold and 10.60-fold, respectively. Among these differentially expressed novel miRNAs, rsa-miRn5 and rsa-miRn17 were only detected in the CK library, whereas three miRNAs (rsa-miRn11, rsa-miRn14, and rsa-miRn19) were detected only in the Cd200 library (Table 6), suggesting that these novel miRNAs might be induced or repressed under Cd stress in radish. The results revealed that these differentially regulated miRNAs may play crucial roles in response to Cd stress in radish.

**Table 6.** The novel cadmium-responsive miRNAs identified from CK and Cd200 libraries in radish

miRNA	CK		Cd200		Fold change log_2_ (Cd200/CK)	Regulation	*P*-value	Significance
	Count	Normalized	Count	Normalized				
rsa-miRn3	65	4.1191	8	0.5926	–2.80	Down-regulated	0	**
rsa-miRn4	1045	66.2231	574	42.5185	–0.64	Down-regulated	0	
rsa-miRn5	5	0.3169	0	0.0010	–8.31	Down-regulated	8.07E-10	**
rsa-miRn11	0	0.0010	242	17.9259	14.13	Up-regulated	3.61E-09	**
rsa-miRn14	0	0.0010	28	2.0741	11.02	Up-regulated	0	**
rsa-miRn15	222	14.0684	34	2.5185	–2.48	Down-regulated	6.73E-10	**
rsa-miRn17	8	0.5070	0	0.0010	–8.99	Down-regulated	1.57E-16	**
rsa-miRn19	0	0.0010	21	1.5556	10.60	Up-regulated	8.15E-07	**

### Degradome sequencing and data summary

**Fig. 4.**
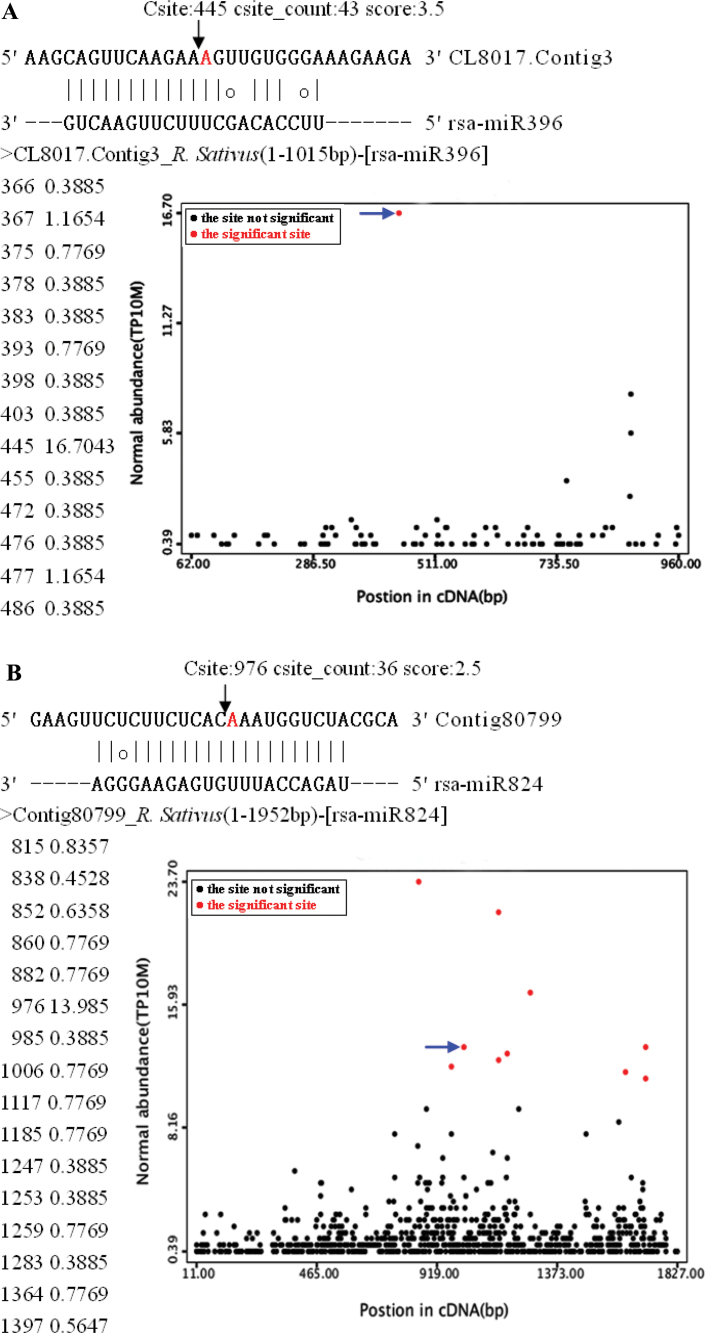

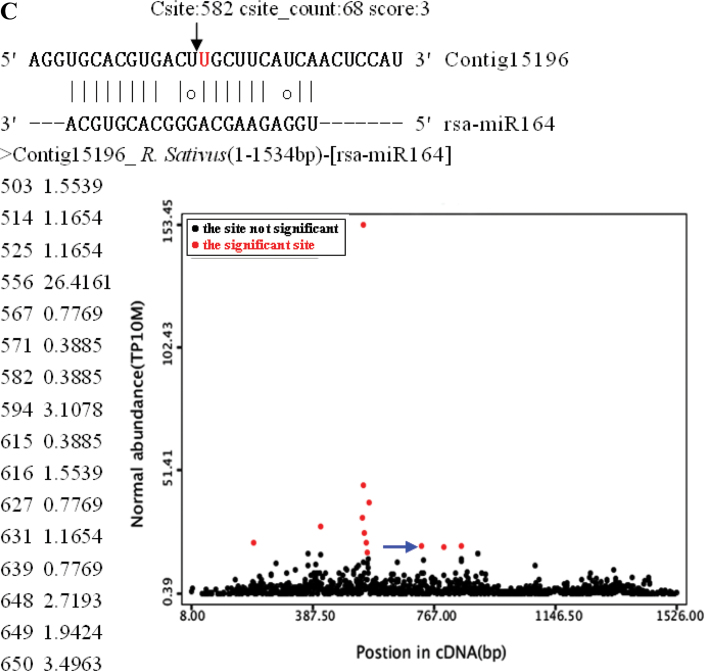
Target plots (t-plots) of miRNA targets in different categories confirmed by degradome sequencing. The normalized signature abundance throughout the length of the indicated transcripts is shown. Representative t-plots for class I (A), class II (B), and class III (C) categories are shown. Blue arrows indicate signatures consistent with miRNA-directed cleavage. The solid lines and dot in miRNA:mRNA alignments indicate matched RNA base pairs and GU mismatch, respectively. On the left of the t-plots, the cleavage sites and normalized signature abundance are shown in the left and right column, respectively.

Although a few miRNAs targets from radish have been previously predicted (Muvva *et al*., 2012; Xu *et al*., 2013), no miRNA targets for radish have yet been experimentally characterized. In this study, the miRNA-cleaved mRNAs in radish were first systematically confirmed using the high-throughput degradome sequencing technology. In total, ~25.87 million raw reads were obtained, which resulted in generating 25 741 860 clean reads representing 8 037 807 unique reads from the mixed degradome library. A total of 6 287 867 unique reads representing 78.2% of the total unique sequences were successfully mapped to the *R. sativus* reference sequences. The CleaveLane pipeline was adopted to identify the sliced targets for the known miRNAs and novel miRNA candidates (Addo-Quaye *et al*., 2009). The abundance of the sequenced tags was plotted on each transcript. The sliced target transcripts were grouped into three categories based on the relative abundance of the degradome signatures at the target mRNA sites (Fig. 4). Based on the previous criteria, category I is defined as abundance at a position where the most abundant reads match the transcript, and with only one maximum on the transcript with more than one raw read at the position. Category II is described as abundance at the position less than the maximum but higher than the median for the transcript and with more than one raw read at the position. Category III is comprised of all the other transcripts sliced by miRNAs.

### Identification and annotation of targets for *radish* miRNAs

**Fig. 5.**
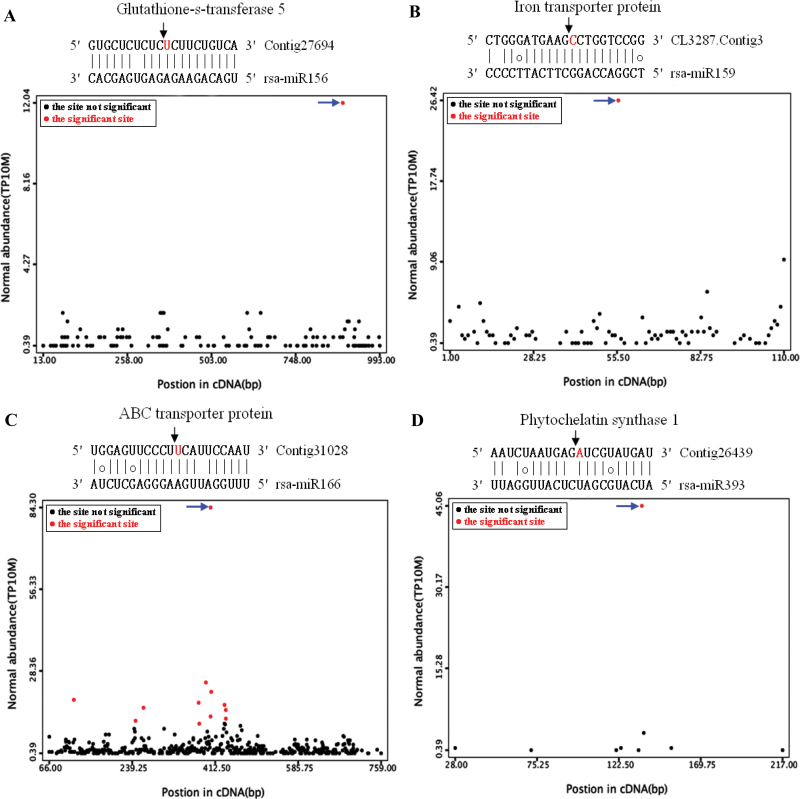
Target plots (t-plots) for a set of Cd-responsive miRNA targets confirmed by degradome sequencing. (A) rsa-miR156, (B) rsa-miR159, (C) rsa-miR166, (D) rsa-miR393. Blue arrows indicate signatures consistent with miRNA-directed cleavage. The solid lines and dot in miRNA:mRNA alignments indicate matched RNA base pairs and GU mismatch, respectively.

Through the degradome sequencing approach, a total of 55 and 16 sliced targets for 17 conserved and seven non- conserved miRNAs were identified, respectively. Among these 71 targets, 45 targets fell into category I, whereas 16 and 10 targets belonged to category II and III, respectively. A number of the identified targets for the known radish miRNAs were transcription factors, such as the auxin response factor (ARF) family, AP2-type transcription factor, NAC (No Apical Meristem) domain transcription factor, zinc finger proteins, squamosa promoter-binding protein (SBP), and TCP family transcription factor (Supplementary Table S6 at *JXB* online), which could play essential regulatory roles in various aspects of plant growth and development. Moreover, many miRNA targets were involved in a wide range of biological processes, including those encoding F-box family protein, protein– protein interaction family proteins, transmembrane protein, and transducin family protein. In addition, a few transcripts targeted by known miRNAs were involved in plant response to biotic and abiotic stresses, such as those encoding leucine-rich repeat (LRR) domain-containing proteins (CL2282.Contig9 and EY922772; miR166 and miR390), heat shock protein (Contig132627, miR396), ABC transporter protein (comp31028, miR159), and iron transporter protein (CL2282.Contig10, miR166) (Fig. 5). These results indicated that the conserved and non-conserved miRNAs might play vital roles in diverse biological processes under Cd stress in radish.

Although the novel miRNAs were sequenced at a relatively lower level as compared with the known miRNAs, their targets were also identified by degradome sequencing analysis. In total, 18 target genes for 10 novel miRNAs were successfully identified (Table 8). Among these candidate targets, 11 targets fell into category I, whereas five and two targets belonged to category II and III, respectively. Compared with the known miRNAs, low splicing frequency targets were found for the novel radish miRNAs, which was consistent with previous studies for some *Brassica* species (M.Y. Xu *et al*., 2012; J.H. Yang *et al*., 2013). Several targets were transcription factors, whereas some other targets appeared to be involved in signal transduction pathways including ARFs, zinc finger proteins, and protein–protein interaction domain family protein (Table 8). Moreover, a few targets could be involved in stress response, such as disease resistance protein (Unigene17426, rsa-miRn11) and cold-inducible plasma membrane protein (Unigene13282, rsa-miRn17). In addition, a small proportion of these novel miRNAs targeted some genes with no functional annotations. These findings suggested that the novel miRNAs may play special roles in some biological and developmental processes under Cd stress in radish. However, no targets were discovered for 12 novel miRNAs in the degradome sequencing data, partly due to the limited number of accessible radish reference sequences.

### qRT-PCR validation of Cd-responsive miRNAs

**Fig. 6.**
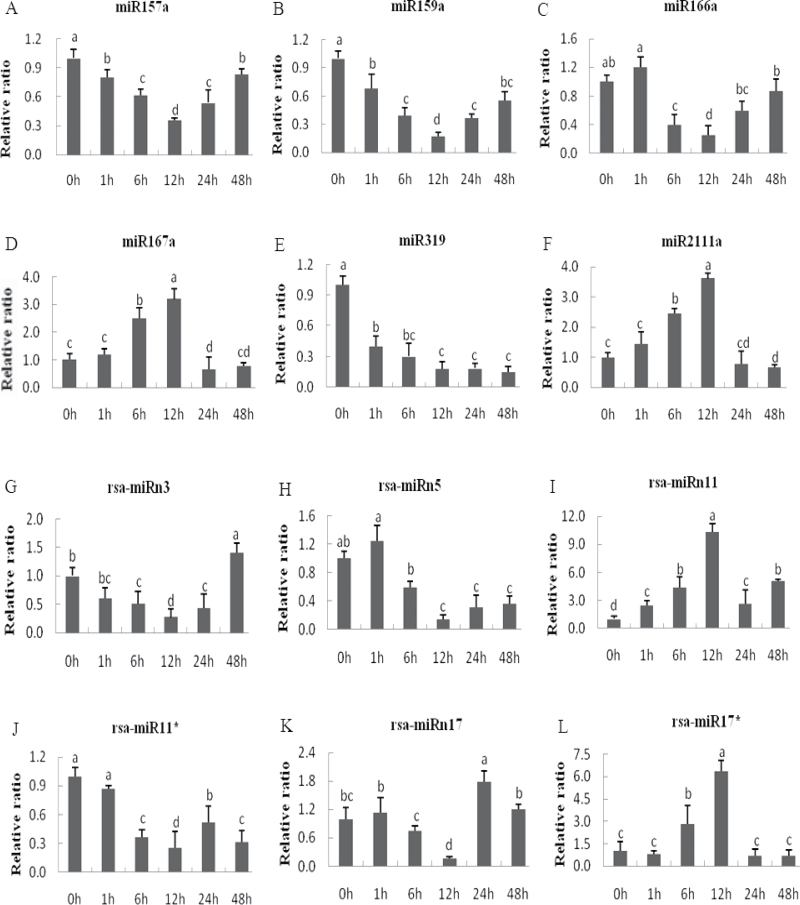
qRT-PCR validation of known Cd-responsive miRNAs in radish. (A–F) and (G–L) Known and novel miRNAs, respectively. Small RNAs (<200 nt) were extracted from radish root treated or not with Cd (0, 1, 6, 12, 24, and 48h). The amount of expression was normalized to the level of 5.8S rRNA. The normalized miRNA levels at 0h were arbitrarily set to 1. Different letters indicate signiﬁcant differences at *P*<0.05 according to Duncan’s multiple range tests.

To conﬁrm the Solexa sequencing results and study the dynamic expression patterns of the Cd-responsive miRNAs at different Cd treatment time points (0, 1, 6, 12, 24, and 48h) in radish, the expression patterns of six known and four novel (two miRNAs with their miRNA*s) Cd-responsive miRNAs were validated by qRT-PCR (Fig. 6). As expected, the qRT-PCR data showed a high degree of agreement with the expression profiles obtained by sRNA sequencing between the CK and Cd200 libraries under Cd treatment at 12h. For known miRNAs, transcripts of miR157a and miR159a were down-regulated, gradually declined at 1, 6, and 12h, and then steadily increased at 24h and 48h. miR166a showed a similar down-regulated expression pattern, except that it slightly increased at 1h of Cd stress. miR167a and miR2111a gradually increased and peaked at 12h, then sharply decreased and remained at a similar level at 24h and 48h. miR319 was down-regulated and remained at an extremely low expression level at all time points (1, 6, 12, 24, and 48h) (Fig. 6).

For these novel Cd-responsive miRNAs, rsa-miRn3 was down-regulated at 1, 6, and 12h, and then reached a relatively high expression level at 48h. rsa-miRn5 was slightly up-regulated at 1h, gradually declined at 6h and 12h, and then reached a relatively low expression level at 24h and 48h. Moreover, the increased expression profiles of rsa-miRn11 and rsa-miRn17 were accompanied by a decrease in rsa-miRn11* and rsa-miRn17*, respectively, indicating that the relative expression patterns of novel miRNAs and their miRNA*s were opposite under Cd stress in radish. However, rsa-miRn11 was expressed at far greater levels than rsa-miRn11*, whereas rsa-miRn17* was expressed at markedly higher levels than rsa-miRn17 (Fig. 6).

## Discussion

Cd is a widespread toxicant and poses a potential significant threat to human health. Recently, high-throughput sequencing has been widely applied to identify comprehensively plant miRNAs responsive to abiotic stress, which could provide powerful information for better understanding of miRNA-mediated regulatory networks in plant response to various stresses (Ruiz-Ferrer and Voinnet, 2009; Khraiwesh *et al*., 2012). Although many functional studies have shown that several known miRNAs were involved in Cd stress in some plant species (Ding *et al*., 2011; Khraiwesh *et al*., 2012; Zhou *et al*., 2012*a*), no study on comprehensive identiﬁcation of Cd-regulated miRNAs and their targets has yet been conducted in radish. In this study, using high-throughput Solexa technology, ~16 and 14 million sRNA raw reads were obtained from the CK and Cd200 sRNA library of radish root, respectively. Due to the unavailability of the full genome sequences of *R. sativus* and limited numbers of GSS and EST sequences in the public NCBI databases, an mRNA transcriptome from radish roots was sequenced for use as a reference sequence, which could provide more valuable information for prediction of candidate pri-miRNA sequences. To the authors’ knowledge, this is the first report on the systematic transcriptome-based identification and characterization of miRNAs and their targets in response to Cd stress in radish.

### Characteristics of *R. sativus* miRNAs responsive to Cd stress

Previous studies have shown that many known miRNAs were highly evolutionarily conserved in the plant kingdom, whereas some other miRNAs were only conserved in one or a few plant species (Martínez *et al*., 2011; Chen *et al*., 2012*a*). In the current study, the majority of conserved miRNAs were represented with high or moderate abundance, whereas several non-conserved miRNAs were represented by <100 reads. This observation revealed that the conserved miRNAs showed relatively higher expression abundance than the non-conserved miRNAs (M.Y. Xu *et al*., 2012; J.H. Yang *et al*., 2013). As expected, the majority of these highly conserved miRNAs in a variety of plant species were also the most abundant classes in the radish root miRNA library (Table 4). Moreover, 13 out of 16 evolutionarily conserved miRNAs were represented by more than one member, whereas the majority of non-conserved miRNAs were represented only by a single member, suggesting that the conserved miRNAs had more family members than the non-conserved miRNAs. These results were consistent with previous reports in *A. thaliana* (Rajagopalan *et al*., 2006), *M. truncaula* (Chen *et al*., 2012b), *B. napus* (Zhou *et al*., 2012*a*), and cucumber (Martínez *et al*., 2011). Similar to previous reports, a large proportion of targets for conserved miRNAs were transcription factors that regulate developmental and growth processes, and fewer were associated with signal transduction and response to environmental stress (Voinnet, 2009; Cuperus *et al*., 2011).

An increasing number of studies have revealed a range of conserved and non-conserved miRNAs that were differentially regulated under Cd stress in rice (Huang *et al*., 2009; Ding *et al*., 2011), *M. truncatula* (Zhou *et al*., 2008), and *B. napus* (Huang *et al*., 2010; Zhou *et al*., 2012*a*), which greatly advanced our understanding of the regulatory roles of plant miRNAs in adaptive response to heavy metal stresses (Khraiwesh *et al*., 2012). In rice, using the miRNA microarray approach, a total of nine miRNA families (miR156, miR162, miR168, miR166, miR171, miR396, miR390, miR1432, and miR444) were down-regulated under Cd stress, whereas only miR528 was significantly up-regulated (Ding *et al*., 2011). In *M. truncatula*, Zhou *et al*. (2008) reported that miR171, miR319, miR393, and miR529 were up-regulated, whereas miR166 and miR398 were down-regulated under Cd stress using a qRT-PCR-based assay. Moreover, seven up-regulated miRNA families (miR158, miR161, miR172, miR398, miR400, miR857, and miR1885) and 10 down-regulated miRNA families (miR159, miR162, miR164, miR171, miR319, miR394, miR395, miR396, miR858, and miR2111) were identified in response to Cd stress in *B. napus* (Zhou *et al*., 2012*a*). In the present study, a total of 22 known miRNAs and eight unique novel miRNAs were identiﬁed to be responsive to Cd stress (Table 6; Supplementary Table S5 at *JXB* online). As expected, most of these previously identified Cd-responsive miRNAs also showed differential regulation under Cd stress. For instance, miR156, miR159, miR166, and miR319 were down-regulated, whereas miR398 and miR857 were up-regulated, which was in agreement with previous studies in *B. napus* or rice (Ding *et al*., 2011; Zhou *et al*., 2012*a*). However, it was found that some previously reported Cd-regulated miRNA families, such as miR161, miR162, and miR172, were not significantly differentially regulated under Cd stress in radish, while miR396a and miR396b were up-regulated under Cd stress. Thus, further functional studies are needed to validate the precise regulatory roles of these miRNAs in plant response to Cd stress.

### miRNA-mediated regulatory networks responsive to Cd stress

Recently, high-throughput degradome sequencing has been shown to be a valuable and efficient approach to validate and characterize target genes of miRNAs in a variety of plant species (German *et al*., 2008; Shamimuzzaman and Vodkin, 2012). Although a wide range of target genes for conserved and non-conserved miRNAs have been previously predicted in several vegetable crops, only a few target genes have been confirmed experimentally (M.Y. Xu *et al*., 2012; Yu *et al*., 2012). This study represents the first transcriptome-based analysis of miRNA targets responsive to Cd stress in radish by degradome sequencing analysis. Consistent with some previous studies, several identified radish miRNA targets belong to a variety of gene families of transcription factors, including ARFs, MYBs, SBPs, AP2-like factors, and NAC-domain proteins (Tables 7, 8), which were found to be highly conserved in other plant species (Martin *et al*., 2010; M.Y. Xu *et al*., 2012). In *Arabidopsis*, miR156 and miR172 were shown to be involved in the regulation of flowering time and floral development by negatively regulating SBP-LIKE (SPLs) proteins and AP2-like factors, respectively (Wu *et al*., 2009). In this study, rsa-miR156 and rsa-miR157 targeted SBP transcription factors, whereas three conserved miRNAs (rsa-miR165, rsa-miR169, and rsa-miR172) targeted AP2-like factors, suggesting that these five conserved miRNAs might play significant roles in regulating flowering time and floral development in radish. In addition, some other targets appeared to be involved in signal transduction, metabolism, disease resistance, and response to environmental stresses.

**Table 7.** Identified targets for known Cd-responsive miRNAs in radish

miRNA	Target ID	Cleavage site	Category	TP100M	Score	Target annotation
miR156	Contig20162	111	I	22.562	1	Squamosa promoter-binding protein
	Contig44431	487	I	16.338	1	Squamosa promoter-binding protein
	Contig27694	126	I	49.403	3	Glutathione *S*-transferase 5 (GST5)
miR157	Rsa#S42048961	357	II	119.034	2	Squamosa promoter-binding protein
miR158	EV566868	183	I	33.065	4	Bax inhibitor-like protein
miR159	EY896930	325	I	192.166	3	MYB family transcription factor
	Rsa#S42022839	268	III	31.898	3	MYB family transcription factor
	Contig31028	106	I	48.236	2.5	ABC transporter family protein
miR166	CL14723.Contig4	798	I	153.655	2.5	HD-ZIP transcription factor
	CL2282.Contig1	888	I	23.729	3	Ring zinc finger protein
	CL3287.Contig3	798	I	23.729	2.5	Iron transporter protein
	CL528.Contig1	699	I	23.729	2.5	Homeodomain-leucine zipper protein
	CL7584.Contig4	888	II	23.729	2.5	Leucine-rich repeat (LRR) domain-containing proteins
	CL14723.Contig1	663	I	243.903	2.5	Peptide chain release factor subunit
	CL2282.Contig8	681	I	23.729	3	Unknown protein
miR169	EX750227	370	II	12.448	3	AP2 domain-containing transcription factor
	CL4257	256	I	27.23	2.5	CCAAT-binding transcription factor
	Contig7182	82	I	56.794	3	CCAAT-binding transcription factor
miR393	Contig26439	256	I	222.508	2	Phytochelatin synthase 1
miR396	CL8017.Contig3	445	I	16.727	3.5	Auxin response factor 8 (ARF8)
	Contig132627	136	III	45.124	3.5	Heat shock protein 90
	Unigene34651	136	III	45.124	3.5	Uncharacterized protein
	FD946893	185	I	27.23	3.5	Hypothetical protein
	Unigene23674	490	I	17.894	4	Drought-stressed protein
	FD986768	531	III	17.894	4	Drought-stressed protein
	FY448028	648	II	16.727	3.5	Uncharacterized protein
	CL8017.Contig1	725	I	16.727	2.5	Transmembrane protein-related
miR408	Unigene1805	426	II	33.454	3	Ascorbate oxidase
	Contig13527	78	I	33.454	2.5	Auxin response factor 6(ARF6)
miR827	EY923470	114	II	24.896	1.5	Ring finger family protein
miR2111	EV552506	301	II	19.061	4	60S ribosomal protein L26-1

Detailed information of targets for all the known miRNAs cab be found in Supplementary Table S6 at *JXB* online.

Categories are defined according to Addo-Quaye *et al*. (2008).

Cleavage site, nucleotide number from the 5′ end of cDNA; TP100M, transcripts per 100 million.

**Table 8.** Candidate targets for novel Cd-responsive miRNAs in radish

miRNA	Target ID	Cleavage site	Category	TP100M	Score	Target annotation
rsa-miRn1	Contig19272	733	I	18.234	4	Auxin response factor 16 (ARF16)
	CL2405.Contig1	113	I	18.234	4	Auxin response factor16 (ARF16)
rsa-miRn3	Contig81072	75	II	35.628	2.5	Protein–protein interaction domain family protein
	Contig11248	461	II	35.628	2.5	Protein–protein interaction domain family protein
rsa-miRn4	Unigene18150	158	I	21.792	3	Zinc finger-containing protein
rsa-miRn8	Contig23480	856	I	31.56	3.5	MYB family transcription factor
	Contig21929	12	II	31.56	3.5	MYB family transcription factor
	Unigene24462	334	II	31.56	3.5	Leucine-rich repeat (LRR) domain-containing proteins
rsa-miRn10	CL10523.Contig1	126	I	10.862	3	AP2 domain-containing transcription factor
rsa-miRn11	Unigene17426	62	I	45.68	3.5	Disease resistance protein
rsa-miRn13	CL1028.Contig2	215	III	19.824	3	CCAAT-binding transcription factor
	Contig2969	83	I	19.824	3	Uncharacterized protein
rsa-miRn17	Unigene13282	256	II	106.52	1	Cold-inducible plasma membrane protein
rsa-miRn19	Contig10826	32	I	86.583	4	Zinc finger-containing protein
	Unigene2104	306	I	86.583	4	Zinc finger-containing protein
	CL3587	78	I	86.583	4	Uncharacterized protein
rsa-miRn21	Contig14638	137	III	42.358	3.5	Unknown protein
	CL6275.Contig2	94	I	42.358	3.5	Calcium-dependent protein kinase

Notably, several key responsive proteins or enzymes for heavy metal uptake and translocation were identified as target transcripts for a few conserved miRNAs. rsa-miR156 targeted a transcript encoding a glutathione *S*-transferase 5 (*GST5*), whereas rsa-miR393 targeted phytochelatin synthase 1 (*PCS1*). In plant cells, one major mechanism related to Cd detoxification is complexation with a range of metal-chelating peptides such as glutathione (GSH) and phytochelatins (PCs), both of which reduced its mobility and sequestered the phytochelatin–metal complexes into the vacuole (Shimo *et al*., 2011; Satoh-Nagasawa *et al*., 2012). Moreover, detoxification of reactive oxygen species (ROS) could be accomplished by synthesis of various antioxidants such as ascorbate, GSH, and glutathione S-transferase (DalCorso *et al*., 2008; Takahashi *et al*., 2011). These findings suggested that rsa-miR156 and rsa-miR393 could be involved in Cd detoxification and mediation via directing regulation of the *GST5* and *PCS1* genes in radish, respectively. Additionally, iron transporter-like protein and ABC transporter protein, which were known to be important transporter proteins for heavy metal uptake and translocation (Zhou *et al*., 2012*a*; Shimo *et al*., 2011), were shown to be targeted by rsa-miR159 and rsa-miR166, respectively. The results indicated that rsa-miR159 and rsa-miR166 might be important participants in Cd uptake and translocation in plants through regulating their corresponding targets. Taken together, the identified miRNA-mediated gene expression of *PCS1, GST5,* iron transporter-like protein, and ABC transporter protein could play critical roles in the regulatory networks of Cd uptake, accumulation, translocation, and detoxification in radish. Therefore, it is reasonable that the miRNA-mediated gene regulation could serve as an important mode of regulatory networks responsive to Cd stress in plants. Nevertheless, further functional analysis of these Cd-responsive miRNAs and their corresponding targets is still necessary for better understanding of the miRNA-mediated regulatory mechanisms underlying plant response to Cd stress.

Although several candidate target transcripts for a proportion of known miRNAs were successfully identified under Cd stress by degradome analysis, there were no detectable sliced target transcripts identified for a few conserved miRNAs (miR162, miR167, miR319, miR391, and miR397) and non-conserved miRNAs (miR857, miR1442, and miR5021). Similar results were found in *M. truncatula* (Zhou *et al*., 2012*b*), soybean (Shamimuzzaman and Vodkin, 2012), cotton (X.Y. Yang *et al*., 2013), and *B. napus* (M.Y. Xu *et al*., 2012; Zhou *et al*., 2012*a*). This may be attributed to the differences in temporal or spatial expression of miRNAs, and their targets may cause insufficient degradation of the target genes, resulting in the levels of these sliced targets being too low to be detected in the degradome (Kawashima *et al*., 2009; M.Y. Xu *et al*., 2012). Furthermore, some plant miRNAs regulate their targets by mRNA cleavage, whereas some other miRNAs may silence their target activity by translational repression (Fabian *et al*., 2010; X.Y. Yang *et al*., 2013). Additionally, because the *R. sativus* full genome sequence is not available yet, the insufficient number of accessible radish reference sequences might limit the identification of targets for both known and novel miRNAs.

In conclusion, this is the first report on the genome-wide identification of novel and Cd-responsive miRNAs and their targets using small RNA sequencing and degradome analysis in radish. A total of 15 known miRNAs and eight novel miRNA families were identified to be responsive to Cd stress. Using degradome sequencing, 18 and 71 targets cleaved by novel and known miRNAs were confirmed in radish for the ﬁrst time. Some target transcripts were functionally predicted to code biotic and abiotic stress-responsive proteins or enzymes. Expression patterns of these differentially regulated miRNAs and their targets were shown to be regulated by Cd stress. These findings could provide new information for further identiﬁcation and characterization of miRNAs in radish, and advance our understanding of the functional characterization of miRNAs and their targets in regulating plant response to Cd stress.

## Supplementary data

Supplementary data are available at *JXB* online.

Figure S1. Venn diagrams for analysis of total (A) and unique (B) small RNAs between CK and Cd200 libraries from radish roots.

Figure S2. The secondary structures of novel *Raphanus sativus* miRNA precursors.

Table S1. qRT-PCR-validated miRNAs and their sequences.

Table S2. Summary of cleaning data produced from CK and Cd200 sRNA libraries of radish.

Table S3. Detailed information of the known miRNAs identified from radish.

Table S4. Detailed information of novel candidate miRNAs identiﬁed from radish.

Table S5. Summary of Cd-responsive miRNAs in radish.

Table S6. Detailed information of targets for known radish miRNAs confirmed by degradome sequencing.

Supplementary Data
