# Supplementary material for: Genome-wide identification and characterization of cadmium-responsive microRNAs and their target genes in radish (Raphanus sativus L.) roots
Source: J Exp Bot. 2013 Sep 7;64(14):4271–87. doi: 10.1093/jxb/ert240 (PMC3808317; doi:10.1093/jxb/ert240)
Supplement: Supplementary Data [file supp_64_14_4271__index.html]

Genome-wide identification and characterization of cadmium-responsive microRNAs and their target genes in radish (Raphanus sativus L.) roots — Genome-wide identification and characterization of cadmium-responsive microRNAs and their target genes in radish (Raphanus sativus L.) roots — Supplementary Data 

# Genome-wide identification and characterization of cadmium-responsive microRNAs and their target genes in radish (*Raphanus sativus* L.) roots

## 

Data files

**Files in this Data Supplement:**

- Supplementary Data - Supplementary Data
