# Supplementary material for: Genome-wide identification and characterization of cadmium-responsive microRNAs and their target genes in radish (Raphanus sativus L.) roots
Source: J Exp Bot. 2013 Sep 7;64(14):4271–87. doi: 10.1093/jxb/ert240 (PMC3808317; doi:10.1093/jxb/ert240)
Supplement: Supplementary Data [file supp_ert240_jexbot099622_file001.pdf]

Supplementary data

Genome-wide identification and characterization of Cadmium-responsive microRNAs and their target genes in radish (*Raphanus sativus* L.) roots

Liang Xu, Yan Wang, Lulu Zhai, Yuanyuan Xu, Liangju Wang, Xianwen Zhu, Yiqin Gong, Rugang Yu, Cecilia Limera, Liwang Liu*

Table S1 qRT-PCR validated miRNAs and their sequences.

Table S2 Summary of cleaning data produced from CK and Cd200 sRNA libraries of radish roots.

Table S3 Detailed information of the known miRNAs identified from radish roots.

Table S4 Detailed information of the novel miRNAs identified from radish roots.

Table S5 Summary of cadmium-responsive miRNAs in radish. The normalized count, fold change and regulated mode were shown.

Table S6 Detailed information of targets for known radish miRNAs confirmed by degradome sequencing.

Fig. S1. Venn diagrams for analysis of total (A) and unique (B) sRNAs between CK and Cd200 libraries from radish roots.

Fig. S2. The secondary structures of novel *Raphanus sativus* miRNA precursors.

Table S1 qRT-PCR validated miRNAs and their sequences.

**miRNA**	**Sequence (5'-3')**
miR157a	UGACAGAAGAGAGUGAGCAC
miR159a	UUUGGAUUGAAGGGAGCUCUA
miR166a	UCGGACCAGGCUUCAUUCCCC
miR167a	UGAAGCUGCCAGCAUGAUCUA
miR319	UUGGACUGAAGGGAGCUCCCU
miR2111	UAAUCUGCAUCCUGAGGUUUA
rsa-miRn3	AAGCUAGAGACUUAAAACAAG
rsa-miRn5	GUGGUGACGGUGGUGGUGCGA
rsa-miRn11	GCUCAAGAAAGCUGUGGGAAA
rsa-miRn11*	UUCCACAGCUUUCUUGAACUU
rsa-miRn17	UUGGACUGAAGGGAGCUCCUU
rsa-miRn17*	GGAGAUUCUUUCAGUCCAAUC
5.8S rRNA	ACGTCTGCCTGGGTGTCACAA

Table S2 Summary of cleaning data produced from CK and Cd200 sRNA libraries of radish roots.

Type	CK		Cd200	
	**Count**	**Percent (%)**	**Count**	**Percent (%)**
total_reads	16,022,680		13,695,577	
high_quality	15,983,864	100%	13,664,651	100%
3' adapter_null	8395	0.05%	7998	0.06%
insert_null	3359	0.02%	3320	0.02%
5' adapter_contaminants	34,062	0.21%	41,085	0.30%
smaller_than_18nt	155,487	0.97%	114,616	0.84%
Poly (A)	3271	0.02%	2382	0.02%
Clean reads	15,779,290	98.72%	13,495,250	98.76%

Table S3 Detailed information of the known miRNAs identified from radish roots.

				miRNA reads		Normalizated read count	
**Family**	**Number**	**Members**	**Sequence (5' to 3')**	CK	Cd400	CK	Cd400
**conserved miRNA**							
miR156	13	miR156a	UGACAGAAGAGAGUGAGCAC	90,678	102,332	5746.3878	7580.1481
		miR156b	UGACAGAAGAGAGUGAGCA	342	266	21.6730	19.7037
		miR156c	UGACAGAAGAGAUUGAGCAC	61	77	3.8657	5.7037
		miR156d	CGACAGAAGAGAGUGAGCAC	46	30	2.9151	2.2222
		miR157a	UUGACAGAAGAUAGAGAGCAC	197,395	67,450	12509.1888	4996.2963
		miR157b	UGACAGAAGAUAGAGAGCAC	2,519	4,186	159.6324	310.0741
		miR157c	UUGACAGAAGAUAGAGAGCACU	425	578	26.9328	42.8148
		miR157d	UUGACAGAAGAUAGAGAGCACUU	193	6	12.2307	0.4444
		miR157e	UUGACAGAAGAUAGAGAGCA	132	167	8.3650	12.3704
		miR157f	GACAGAAGAUAGAGAGCAC	49	97	3.1052	7.1852
		miR157g	UUGACAGAAGAUAGAGCAC	18	51	1.1407	3.7778
		miR157h	UUACAGAAGAUAGAGAGCAC	10	28	0.6337	2.0741
		miR157i	UUGACAGAAGAUAGAGAGCACUC	14	26	0.8872	1.9259
miR158	9	miR158a	UCCCAAAUGUAGACAAAGCA	114,171	2,352	7235.1711	174.2222
		miR158b	UCCCAAAUGUAGACAAAGCAU	401	284	25.4119	21.0370
		miR158c	UUCCCAAAUGUAGACAAAGCA	273	172	17.3004	12.7407
		miR158d	CUCCCAAAUGUAGACAAAGCA	42	23	2.6616	1.7037
		miR158e	UCCCAAUGUAGACAAAGCA	22	12	1.3942	0.8889
		miR158f	CCCCAAAUGUAGACAAAGCA	22	20	1.3942	1.4815
		miR158g	UCCCAAAUGUAGACAAAGCAC	26	19	1.6477	1.4074
		miR158h	UCCCAAAUGUAGACAAAGCAUA	8	11	0.5070	0.8148
		miR158i	UCCCAAAUGUAGACAAAGCAG	7	11	0.4436	0.8148

miR159	4	miR159a	UUUGGAUUGAAGGGAGCUCUA	1,883	1,081	119.3283	80.0741
		miR159b	UUUGGAUUGAAGGGAGCUUA	171	124	10.8365	9.1852
		miR159c	UUUGGAUUGAAGGGAGCUCU	18	16	1.1407	1.1852
		miR159d	UUUGGAUUGAAGGGAGUCCUA	9	12	0.5703	0.8889
miR160	2	miR160a	AUGCCUGGCUCCCUGUAUGCCA	9	73	0.5703	5.4074
		miR160b	UGCCUGGCUCCCUGUAUGCCA	36	49	2.2814	3.6296
miR162	2	miR162a	UCGAUAAACCUCUGCAUCCAG	1,831	723	116.0330	53.5556
		miR162b	UCGAUAAAUUUCUGCAUCCAG	30	19	1.9011	1.4074
miR164	8	miR164a	UGGAGAAGCAGGGCACGUGCA	52,204	34,819	3308.2383	2579.1852
		miR164b	UGGAGAAGCAGGGCACGUCA	15	16	0.9506	1.1852
		miR164c	UGGAGAAGCAGGGCACGUGCAAU	37	15	2.3447	1.1111
		miR164d	UGGAGAAGCAGGGCACGUGCAAA	9	14	0.5703	1.0370
		miR164e	UGGAGAAGCAGGGCACGUGCAC	853	681	54.0558	50.4444
		miR164f	UGGAGAAGCAGGGCACGUGCG	1,442	584	91.3815	43.2593
		miR164g	UGGAGAAGCAGGGCACGUGCAA	219	121	13.8783	8.9630
		miR164h	GGAGAAGCAGGGCACGUGCA	153	41	9.6958	3.0370
miR165	4	miR165a	UCGGACCAGGCUUCAUCCCCC	6,903	1,676	437.4525	124.1481
		miR165b	UCGGACCAGGCUUCAUCAUCC	32	11	2.0279	0.8148
		miR165c	UCGGACCAGGCUUUAUCCCCC	27	10	1.7110	0.7407
		miR165d	UCGGACCAGGCUUCAUGCCCC	24	10	1.5209	0.7407
miR166	11	miR166a	UCGGACCAGGCUUCAUUCCCC	187,287	56,498	11868.6312	4185.0370
		miR166b	UCGGACCAGGGUUCAUUCCCC	1,614	440	102.2814	32.5926
		miR166c	UCGGAUUAGGCUUCAUUCCCC	191	69	12.1039	5.1111
		miR166d	UCGGACUAGGCUUCAUUCCCC	326	79	20.6591	5.8519
		miR166e	UCGGACCAGGCUCAUUCCCC	45	16	2.8517	1.1852
		miR166f	UCGGACCAGACUUCAUUCCCC	40	5	2.5349	0.3704

		miR166g	UCGGACCAGGCUUAUUCCCC	67	15	4.2459	1.1111
		miR166h	UCGGACCAUGCUUCAUUCCCC	26	14	1.6477	1.0370
		miR166i	UCGGACCAGGCUCCAUUCCCC	23	13	1.4575	0.9630
		miR166j	UCGGACCAGGUUUCGUUCCCC	24	10	1.5209	0.7407
		miR166k	UCGGACCAGGCUUCAUUCCCA	26	10	1.6477	0.7407
miR167	7	miR167a	UGAAGCUGCCAGCAUGAUCUA	2,226	12,401	141.0646	918.5926
		miR167b	UGAAGCUGCCAGCAUGAUCU	101	17	6.4005	1.2593
		miR167c	UGAAGCUGCCAGCGUGAUCU	12	15	0.7605	1.1111
		miR167d	AAGCUGCCAGCAUGAUCUUGU	10	12	0.6337	0.8889
		miR167e	UAAGCUGCCAGCAUGAUCUUGU	247	295	15.6527	21.8519
		miR167f	UAAGCUGCCAGCAUGAUCUUGUC	10	23	0.6337	1.7037
		miR167g	UGAAGCUGCCAGCAUGAUCUGG	22	31	1.3942	2.2963
miR168	3	miR168a	UCGCUUGGUGCAGGUCGGGAA	61,145	53,201	3874.8416	3940.8148
		miR168b	UCGCUUGGUGCAGGUCGGGAAC	11,669	7,855	739.4804	581.8519
		miR168c	UCGCUUGGUGCAGGUCGGGAAA	15	28	0.9506	2.0741
miR169	4	miR169a	CAGCCAAGGAUGACUUGCCGG	605	558	38.3397	41.3333
		miR169b	CAGCCAAGGAUGACUUGCCGGU	19	8	1.2041	0.5926
		miR169c	UGAGCCAAGGAUGAUUUGCCG	98	21	6.2104	1.5556
		miR169d	UGAGCCAAGGAUGACUUGCCG	61	17	3.8657	1.2593
miR172	3	miR172a	AGAAUCUUGAUGAUGCUGCAU	1,112	1,705	70.4689	126.2963
		miR172b	AGAAUCUUGAUGAUGCUGCAG	845	159	53.5488	11.7778
		miR172c	GGAAUCUUGAUGAUGCUGCAU	146	69	9.2522	5.1111
miR319	1	miR319	UUGGACUGAAGGGAGCUCCCU	7241	472	458.8720	34.9630
miR390	3	miR390a	AAAGCUCAGGAGGGAUAGCGCC	132	24	8.3650	1.7778
		miR390b	AAGCUCAGGAGGGAUAGCGCC	11,848	3,651	750.8238	270.4444
		miR390c	AGCUCAGGAGGGAUAGCGCC	13	73	0.8238	5.4074

miR391	2	miR391a	UUCGCAGGAGAGAUAGCGCCA	2,131	1,208	135.0444	89.4815
		miR391b	UUCGCAGGAGAGAUAGCGCCU	26	20	1.6477	1.4815
miR393	2	miR393a	AUCAUGCGAUCUCAUUGGAUU	22	146	1.3942	10.8148
		miR393b	CAUGCGAUCUCAUUGGAUUCAA	8	78	0.5070	5.7778
miR395	4	miR395a	CCACUGAAGUGUUUGGGGGAACUC	7	11	0.4436	0.8148
		miR395b	ACUGAAGUGUUUGGGGGAACU	2	11	0.1267	0.8148
		miR395c	CUGAAGUGUUUGGGGGAACUC	139	302	8.8086	22.3704
		miR395d	CUGAAGUGUUUGGGGGGACUC	62	153	3.9290	11.3333
miR396	2	miR396a	UUCCACAGCUUUCUUGAACUG	892	21577	56.5272	1598.2963
		miR396b	UUCCACAGCUUUCUUGAACUU	58	176	3.6755	13.0370
miR397	1	miR397	UCAUUGAGUGCAGCGUUGAUG	3,252	3,013	206.0837	223.1852
miR398	1	miR398	GUGUUCUCAGGUCACCCCUGC	164	753	10.3929	55.7778
miR399	2	miR399a	UGCCAAAGGAGAGUUGCCCUG	20	29	1.2674	2.1481
		miR399b	UGCCAAAGGAGAUUUGCCCGG	3	26	0.1901	1.9259
miR408	5	miR408a	AUGCACUGCCUCUUCCCUGGC	4,682	3,369	296.7047	249.5556
		miR408b	AUGCACUGCCUCUUUUCUGGC	413,125	18,474	26180.2915	1368.4444
		miR408c	AUGCACUGCCUCUUUCCUGGC	5	12	0.3169	0.8889
		miR408d	AUGCACUGCCUCUUCCCUGGCU	21	11	1.3308	0.8148
		miR408e	AUGCACUGCCUCUUCCCUGGA	10	23	0.6337	1.7037
**non-conserved miRNA**							
miR403	3	miR403a	UUAGAUUCACGCACAAACUCG	1,115	627	70.6591	46.4444
		miR403b	UUAGAUUCACGCACAAACU	34	19	2.1546	1.4074
		miR403c	UUAGAUUCACGCACAAACUC	19	13	1.2041	0.9630
miR824	2	miR824a	UAGACCAUUUGUGAGAAGGGA	1,170	558	74.1445	41.3333
		miR824b	AGACCAUUUGUGAGAAGGGA	8	10	0.5070	0.7407
miR825	2	miR825a	UUUCAAGAGAGGUGCAUGAACU	100	33	6.3371	2.4444

		miR825b	UUUUCAAGAGAGGUGCAUGAAC	15	9	0.9506	0.6667
miR827	2	miR827a	UUAGAUGACCAUCAACAAAUA	6	104	0.3802	7.7037
		miR827b	UAGAUGACCAUCAACAAAUAGC	30	368	1.9011	27.2593
miR854	1	miR854a	GAGAGGAUGGGAGGACGACG	485	145	30.7351	10.7407
miR857	1	miR857a	AUUUUGUAUGUUGAAGGUGUA	1,598	3162	101.2674	234.2222
miR1023	1	miR1023a	AGGAAACUCUGGUGGAGGCAU	5	8	0.3169	0.5926
miR1442	1	miR1442a	AUUGAUAGUAGAGAUGUGUA	183	26	11.5970	1.9259
miR2111	2	miR2111a	UAAUCUGCAUCCUGAGGUUUA	6	32	0.3802	2.3704
miR5021	1	miR5021a	GAGGAAGAAGAAGAAGGACAA	15	76	0.9506	5.6296

Table S4 Detailed information of the novel miRNAs identified from radish roots.

miRNA family	Count		Normalized reads		Sequence (5'–3')	Arm	Size	LP (nt)	MFE (kcal/mol)	miRNA location
	CK	Cd400	CK	Cd400						
rsa-miRn1	8263	9468	523.6375	701.3333	UCGCUUGGUGCAGGUCGGGAA	5'	21	141	-72.20	EY910368
rsa-miRn1*	53	146	3.3587	10.8148	CCCGCCUUGUAUCAAGUGAAU	3'	21	141	-72.20	EY910368
rsa-miRn2	1524	4358	96.5779	322.8148	UGAAGCUGCCAGCAUGAUCUA	5'	21	118	-48.00	EX889075
rsa-miRn2*	2	14	0.1267	1.0370	GAUCAUGUUUGCAGUUUCACC	3'	21	118	-48.00	EX889075
rsa-miRn3	65	8	4.1191	0.5926	AAGCUAGAGACUUAAAACAAG	5'	21	139	-23.95	EW723459
rsa-miRn3*	17	2	1.0773	0.1481	UGUUUAAUAAGUAUGGUUGAG	3'	21	139	-23.95	EW723459
rsa-miRn4	1045	574	66.2231	42.5185	GCGUAUGAGGAGCCAAGCAUA	3'	21	106	-49.30	EX888945
rsa-miRn4*	132	36	8.3650	2.6667	AUGCCUGGCUCCCUGUAUGCC	5'	21	106	-49.30	EX888945
rsa-miRn5a	5	0	0.3169	0.0010	GUGGUGACGGUGGUGGUGCGA	3'	21	99	-36.40	FD951238
rsa-miRn5a*	0	1	0.0010	0.0741	GCGCCGCCAUGGAAGUCGCCAC	5'	22	99	-36.40	FD951238
rsa-miRn5b	5	0	0.3169	0.0010	GUGGUGACGGUGGUGGUGCG	3'	21	94	-36.40	FD955132
rsa-miRn5b*	0	1	0.0010	0.0741	GCGCCGCCAUGGAAGUCGCCAC	5'	22	94	-36.40	FD955132
rsa-miRn6	3972	2741	251.7110	203.0370	CAGGGAACAAGCAGAGCAUGG	5'	21	110	-46.10	EW713854
rsa-miRn6*	899	658	56.9708	48.7407	AUGCACUGCCUCUUCCCUGGC	3'	21	110	-46.10	EW713854
rsa-miRn7	58	362	3.6755	26.8148	AUAUACUGAAGUUUAUACUCU	5'	21	208	-37.00	EY928450
rsa-miRn7*	2	34	0.1267	2.5185	AUCAUAAAAUCUUCAUUAUCUAG	3'	23	208	-37.00	EY928450
rsa-miRn8	1137	563	72.0532	41.7037	GUAUGAGGAGCCAAGCAUAU	3'	21	105	-46.60	EY928785
rsa-miRn8*	145	53	9.1888	3.9259	AUGCCUGGCUCCCUGUAUGCC	5'	21	105	-46.60	EY928785
rsa-miRn9a	36	25	2.2814	1.8519	GUACGACGAAGAUGAGCCGACAC	5'	23	110	-19.60	FY430263
rsa-miRn9a*	4	1	0.2535	0.0741	GUCGCACUCAUCAUCGGCGUCACG	3'	24	110	-19.60	FY430263
rsa-miRn9b	36	25	2.2814	1.8519	GUACGACGAAGAUGAGCCGACAC	5'	23	75	-23.00	FY450534
rsa-miRn9b*	4	1	0.2535	0.0741	GUCGCACUCAUCAUCGGCGUCACG	3'	24	75	-23.00	FY450534
rsa-miRn9c	22	14	1.3942	1.0370	GUACGACGAAGAUGAGCCGACAC	5'	23	84	-20.70	CL10123.Contig3

rsa-miRn9c*	6	1	0.3802	0.0741	GUCGCACUCAUCAUCGGCGUCACGG	3'	25	84	-20.70	CL10123.Contig3
rsa-miRn10	378	452	23.9544	33.4815	UGGAGGCAGCGGUUCAUCGAUC	5'	22	140	-45.70	FD536486
rsa-miRn10*	1842	528	116.7300	39.1111	UCGAUAAACCUCUGCAUCCAG	3'	21	140	-45.70	FD536486
rsa-miRn11	0	242	0.0010	17.9259	GCUCAAGAAAGCUGUGGGAAA	3'	21	147	-39.04	FD570951
rsa-miRn11*	0	72	0.0010	5.3333	UUCCACAGCUUUCUUGAACUU	5'	21	147	-39.04	FD570951
rsa-miRn12	59	0	3.7389	0.0010	AAACUGCCUAAACAAACAUAUC	5'	22	171	-40.44	FD578260
rsa-miRn12*	11	0	0.6971	0.0010	UAUGCUUUUUAGGCAGUUUCA	3'	21	171	-40.44	FD578260
rsa-miRn13	827	2326	52.4081	172.2963	GCUGGAGGCAGCGGUUCAUCGAUC	5'	24	142	-46.80	FD938184
rsa-miRn13*	132	168	8.3650	12.4444	UCGAUAAACCUCUGCAUCCAGCG	3'	23	142	-46.80	FD938184
rsa-miRn14a	0	28	0.0010	2.0741	AGAUGACAGUGAGGCUUCUUA	5'	21	108	-18.20	EV525816
rsa-miRn14a*	0	1	0.0010	0.0741	AGAGCUUUAUGGUUUCUCU	3'	19	108	-18.20	EV525816
rsa-miRn14b	0	28	0.0010	2.0741	AGAUGACAGUGAGGCUUCUUA	5'	21	113	-19.50	EW731106
rsa-miRn14b*	0	1	0.0010	0.0741	AGAGCUUUAUGGUUUCUCU	3'	19	113	-19.50	EW731106
rsa-miRn14c	0	28	0.0010	2.0741	AGAUGACAGUGAGGCUUCUUA	5'	21	113	-18.50	FD988883
rsa-miRn14c*	0	1	0.0010	0.0741	AGAGCUUUAUGGUUUCUCU	3'	19	113	-18.50	FD988883
rsa-miRn14d	0	28	0.0010	2.0741	AGAUGACAGUGAGGCUUCUUA	5'	21	113	-19.50	EY898311
rsa-miRn14d*	0	1	0.0010	0.0741	AGAGCUUUAUGGUUUCUCU	3'	19	113	-19.50	EY898311
rsa-miRn15	222	34	14.0684	2.5185	CCCGCCUUGCAUCAACUGAAU	3'	21	137	-66.30	FD955742
rsa-miRn15*	19	0	1.2041	0.0010	UCGCUUGGUGCAGUUCGGGAC	5'	21	137	-66.30	FD955742
rsa-miRn16	12154	25732	770.2155	1906.0741	GCUUGGUGCAGGUCGGGACUA	5'	21	142	-73.60	FD971386
rsa-miRn16*	1223	2053	77.5032	152.0741	CGCCUUGCAUCAACUGAAUCG	3'	21	142	-73.60	FD971386
rsa-miRn17	8	0	0.5070	0.0010	UUGGACUGAAGGGAGCUCCUU	3'	21	201	-86.70	FY434434
rsa-miRn17*	1	0	0.0634	0.0010	GGAGAUUCUUUCAGUCCAAUC	5'	21	201	-86.70	FY434434
rsa-miRn18	1721	4823	109.0621	357.2593	CCCGCCUUGCAUCAACUGAAU	3'	21	144	-56.70	FY447875
rsa-miRn18*	14	156	0.8872	11.5556	UCGCUUGAUGCAGGUCGGGAC	5'	21	144	-56.70	FY447875
rsa-miRn19	0	9	0.0010	0.6667	AUGGAUGUAUGAUAUGAUGGA	3'	21	136	-41.40	EX886790

rsa-miRn19*	0	3	0.0010	0.2222	UAUUAAUCUGUAUGUUGUUUC	5'	21	136	-41.40	EX886790
rsa-miRn20	10	27	0.6337	2.0000	GGAAUGUUGUUUGGCUCGAAG	3'	21	72	-20.00	CL13700.Contig2
rsa-miRn20*	0	1	0.0010	0.0741	UCGACGGGAAGGGGCUUUCUCU	5'	22	72	-20.00	CL13700.Contig2
rsa-miRn21	36853	53357	2335.4246	3952.3704	UCGGACCAGGCUUCAUUCCCC	3'	21	133	-67.20	CL9669.Contig1
rsa-miRn21*	196	240	12.4208	17.7778	GGAAUGUUGUCUGGCUCGAGG	5'	21	133	-67.20	CL9669.Contig1
rsa-miRn22	2638	3280	167.1736	242.9630	AAGCUGCCAGCGUGAUCUUAAC	5'	22	101	-41.80	CL5433.Contig1
rsa-miRn22*	875	1143	55.4499	84.6667	UCAGAUCAUGUGGCAGUUUCA	3'	21	101	-41.80	CL5433.Contig1

Table S5 Summary of cadmium-responsive miRNAs in radish. The normalized count, fold change and regulated mode were shown.

Family	miRNA name	miRNA reads		Normalized reads		fold change	regulate	p-value	sig-lable
		CK	Cd400	CK	Cd400	log2 (Cd400/CK)			
156	miR157a	197,395	67,450	12509.1888	4996.2963	-1.32	down-regulated	1.31E-48	**
	miR157d	193	6	12.2307	0.4444	-4.78	down-regulated	4.03E-06	
158	miR158a	114,171	2,352	7235.1711	174.2222	-5.38	down-regulated	7.81E-105	
	miR158c	273	172	17.3004	12.7407	-0.44	down-regulated	0	
	miR158e	22	12	1.3942	0.8889	-0.65	down-regulated	1.68E-11	
159	miR159a	1,883	1,081	119.3283	80.0741	-0.58	down-regulated	0	
166	miR166a	187,287	56,498	11868.6312	4185.0370	-1.50	down-regulated	1.77E-21	**
	miR166d	326	79	20.6591	5.8519	-1.82	down-regulated	0	**
	miR166f	40	5	2.5349	0.3704	-2.77	down-regulated	6.92E-08	**
167	miR167a	2,226	12,401	141.0646	918.5926	2.70	up-regulated	3.52E-27	**
	miR167d	1	12	0.0634	0.8889	3.81	up-regulated	0	**
169	miR169b	19	8	1.2041	0.5926	-1.02	down-regulated	1.12E-26	
319	miR319	7241	472	458.8720	34.9630	-3.71	down-regulated	8.40E-31	**
393	miR393a	22	146	1.3942	10.8148	2.96	up-regulated	5.29E-73	**
396	miR396a	892	21,577	56.5272	1598.2963	4.82	up-regulated	1.84E-11	**
	miR396b	58	176	3.6755	13.0370	1.83	up-regulated	2.47E-09	**
398	miR398	164	753	10.3929	55.7778	2.42	up-regulated	0	**
408	miR408b	413,125	18,474	26180.2915	1368.4444	-4.26	down-regulated	3.30E-51	
827	miR827a	6	104	0.3802	7.7037	4.34	up-regulated	2．35E−11	**
857	miR857a	1598	3162	101.2674	234.2222	1.21	up-regulated	3.16E-10	**
1442	miR1442a	183	26	11.5970	1.9259	-2.59	down-regulated	8.43E-17	**
2111	miR2111a	6	32	0.3802	2.3704	2.64	up-regulated	9.12E-69	**
rsa-miRn3	rsa-miRn3	65	8	4.1191	0.5926	-2.80	down-regulated	0	**

rsa-miRn4	rsa-miRn4	1045	574	66.2231	42.5185	-0.64	down-regulated	0	
rsa-miRn5	rsa-miRn5a	5	0	0.3169	0.0010	-8.31	down-regulated	8.07E-10	**
rsa-miRn11	rsa-miRn11	0	242	0.0010	17.9259	14.13	up-regulated	3.61E-09	**
rsa-miRn14	rsa-miRn14a-d	0	28	0.0010	2.0741	11.02	up-regulated	0	**
rsa-miRn15	rsa-miRn15	222	34	14.0684	2.5185	-2.48	down-regulated	6.73E-10	**
rsa-miRn17	rsa-miRn17	15	0	0.9506	0.0010	-9.89	down-regulated	1.57E-16	**
rsa-miRn19	rsa-miRn19	0	9	0.0010	0.6667	9.38	up-regulated	8.15E-07	**

Table S6 Detailed information of targets for known radish miRNA confirmed by degradome sequencing.

miRNA family	Target gene	Cleavage site	Category	Abundance	TP100M	Percentage (%)	Score	Target annotation
**conserved**								
miR156	Contig20162	111	I	58	22.562	29	1	Squamosa promoter-binding protein
	Contig44431	487	I	42	16.338	37.5	1	Squamosa promoter-binding protein
	Contig27694	126	I	127	49.403	13.62	3	Glutathione s-transferase 5 (GST5)
miR157	Rsa#S42048961	357	II	306	119.034	52.4	2	Squamosa promoter-binding protein
miR158	EV566868	183	I	85	33.065	10.29	4	Bax inhibitor-like protein
miR159	EY896930	325	I	494	192.166	53.76	3	MYB family transcription factor
	Rsa#S42022839	268	III	82	31.898	16.25	3	MYB family transcription factor
	Contig31028	106	I	124	48.236	18.62	2.5	ABC transporter family protein
miR160	FD956077	581	I	33	12.837	1.16	4	Calmodulin (CaM)-like protein
	Contig71254	42	I	113	43.957	72.9	4	Auxin response factor 8(ARF8)
	EY946741	592	I	33	12.837	10.74	4	Calmodulin (CaM)-like protein
	EV538567	719	II	33	12.837	12.27	4	Calmodulin (CaM)-like protein
	Contig1694	192	III	113	43.957	22.1	4	Auxin response factor 6(ARF6)
miR164	FD559847	712	I	38	14.782	10.18	3	Unknown protein
	FY432950	754	I	333	129.537	59.15	3.5	NAC domain containing protein 6 (NAC6)
	CL63.Contig1	300	I	160	62.24	52.63	1.5	NAM (No apical meristem)-like protein
	FD971021	197	II	68	26.452	6.24	3	NAM (No apical meristem)-like protein
	Contig15196	582	I	68	26.452	18.68	3	NAM (No apical meristem)-like protein
	Contig31437	203	I	333	129.537	70.11	3.5	NAC domain containing protein
	EX770141	140	I	68	26.452	0.68	3	Uncharacterized protein
miR165	CL2282.Contig13	681	II	61	23.729	0.54	2.5	AP2 domain-containing transcription factor
	CL14723.Contig3	888	I	395	153.655	28.62	2.5	Auxin Signaling F-Box 1 (AFB1)
	Contig4159	50	III	627	243.903	19.61	2.5	Auxin Signaling F-Box 2 (AFB2)

miR166	CL1746	798	I	395	153.655	28.69	2.5	HD-ZIP transcription factor
	CL2282.Contig1	888	I	61	23.729	5.57	3	Ring zinc finger protein
	CL3287.Contig3	798	I	61	23.729	5.59	2.5	Iron transporter-like protein
	CL528.Contig1	699	I	61	23.729	5.52	2.5	Homeodomain-leucine zipper protein
	CL7584.Contig4	888	II	61	23.729	5.58	2.5	Leucine-rich repeat (LRR) domain-containing proteins
	CL14723.Contig1	663	I	627	243.903	28.74	2.5	Peptide chain release factor subunit
	CL2282.Contig8	681	I	61	23.729	0.54	3	Unknown protein
miR168	Contig46296	86	II	176	68.464	88.89	3.5	S-adenosyl-l-methionine-dependent protein
miR169	EX750227	370	II	32	12.448	14.28	3	AP2 domain-containing transcription factor
	CL4257	256	I	70	27.23	8.64	2.5	CCAAT-binding transcription factor
	Contig7182	82	I	146	56.794	17.82	3	CCAAT-binding transcription factor
miR172	FD572123	290	II	28	10.892	13.59	1.5	Floral homeotic protein APETALA 2
	Contig8716	1349	I	28	10.892	4.53	1.5	Ethylene-responsive transcription factor
	EY906836	497	I	28	10.892	8.16	1.5	Floral homeotic protein APETALA 2
	comp22360	136	I	28	10.892	9.62	3	Disease resistance protein
	EW732550	376	III	164	63.796	48.66	0.5	AP2 domain-containing transcription factor
miR390	EY922772	434	II	47	18.283	2.66	4	Leucine-rich repeat (LRR) domain-containing proteins
miR393	Contig26439	256	I	572	222.508	21.64	2	Phytochelatin synthase 1
miR395	FY437914	391	I	756	294.084	17.89	2.5	ATP sulfurylase like protein
miR396	CL8017.Contig3	445	I	43	16.727	18.3	3.5	Hypothetical protein
	Contig132627	136	III	116	45.124	85.93	3.5	Heat shock protein 90
	Unigene34651	136	III	116	45.124	85.93	3.5	Uncharacterized protein
	FD946893	185	I	70	27.23	0.25	3.5	Hypothetical protein
	Unigene23674	490	I	46	17.894	41.44	4	Drought-stressed protein
	FD986768	531	III	46	17.894	53.49	4	Drought-stressed protein
	FY448028	648	II	43	16.727	32.33	3.5	Uncharacterized protein

	CL8017.Contig1	725	I	43	16.727	15.64	2.5	Transmembrane protein-related
miR399	Contig19272	593	I	53	20.617	19.92	2.5	40S ribosomal protein S10-like
	EX890146	18	I	27	10.503	11.02	3.5	Protein–protein interaction family proteins
	FD560927	46	III	27	10.503	9.64	4	Protein–protein interaction family proteins
miR408	Unigene1805	426	II	86	33.454	12.53	3	Ascorbate oxidase
	Contig13527	78	I	86	33.454	16.36	2.5	Auxin response factor 6(ARF6)
**non-conserved**								
miR403	EX749374	538	III	374	145.486	23.51	2	Putative argonaute family protein
	Contig3071	3424	I	374	145.486	17.49	1	Putative argonaute family protein
	Unigene28750	3432	I	374	145.486	17.82	1	Uncharacterized protein
	Rsa#S41987411	553	I	374	145.486	23.85	1	Transducin family protein
miR824	Contig80799	976	II	36	14.004	38.3	2.5	MADS-box transcription factor
	Contig11744	689	I	100	38.9	18.18	0.5	MADS-box transcription factor
miR825	EV536169	680	III	64	24.896	3.64	3	Ethylene-forming enzyme protein
	EV567411	359	I	64	24.896	3.81	3	Ethylene-forming enzyme protein
miR827	EY923470	114	II	64	24.896	31.22	1.5	Ring finger family protein
miR854	CL4761.Contig1	516	I	77	29.953	0.87	4	Zinc finger-containing protein
	EX895002	167	I	1310	509.59	18.39	4	Mitochondrial substrate carrier family protein
	FY449153	234	III	37	14.393	7.99	4	Peptide chain release factor subunit
	EV535403	173	I	1308	508.812	36.1	2	Zinc finger-containing protein
	EY914658	59	II	2453	954.217	26.99	4	Zinc finger-containing protein
miR1023	EY938475	166	I	36	14.004	30.82	4	Glycosyltransferase subunit DAD1
miR2111	EV552506	301	II	49	19.061	2.09	4	60S ribosomal protein L26-1

Cleavage site: Nucleotide number from 5’ end of cDNA. TP100M: Transcripts per 100 million. Percentage: Percentage of cleavage at the expected site (%).

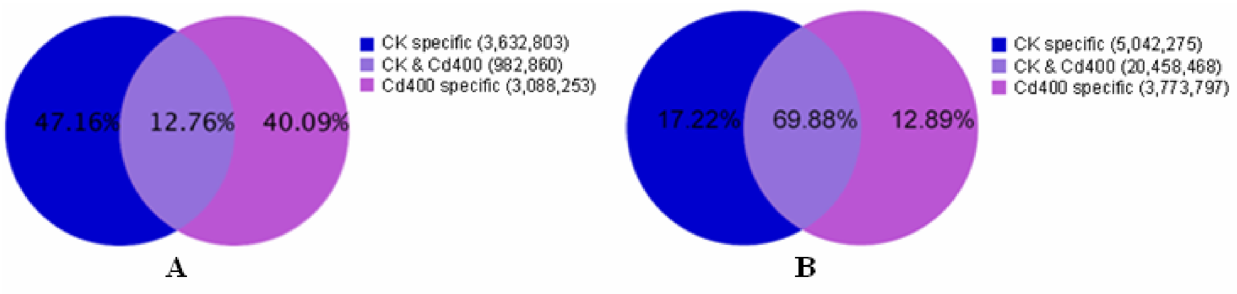

Fig. S1 Venn diagrams for analysis of total (A) and unique (B) sRNAs between CK and Cd200 libraries from radish roots.

Fig. S2 The secondary structures of novel *Raphanus sativus* miRNA precursors. The mature miRNAs are in red and miRNA*s are in blue. (“.” represent base mismatches, “(” represent base matches).

rsa-miRn1

UAUGUUGCGGCGGCUCUGAUUCGCUUGGUGCAGGUCGGGAACUGAUUGGCUGACACAGCCACGUGGGUUUUCAUGGUUG
GCUCGUGAGCAGGGAUCGGAUCCCGCCUUGUAUCAAGUGAAUCCGAGUCCGACGUGAAAGAU
....(..((.((((((.(((((((((((((((((.(((((.((((((.(((.((..((((((((((....)))))..))
))).)).)))...)))))).))))).))))))))))))))))).))).))).))..).....
********************UCGCUUGGUGCAGGUCGGGAA**************************************
****
****
*********************CCCGCCUUGUAUCAAGUGAAU********************

rsa-miRn2

AGGCAUCUGAUGAAGCUGCCAGCAUGAUCUAAUUAACUUUCUUUCUCCGUUUGUUUUCAUGACGAUGGAAAGAGAUGAG
UGUAGAUUAGAUCAUGUUUGCAGUUUCACCCGUUGACUA
.((((.(.(.(((((((((.((((((((((((((.(((((((((.((((((.(((.....))))))))).))))).)))
)...)))))))))))))).))))))))).).).)).)).
**********UGAAGCUGCCAGCAUGAUCUA**
****
****
*********GAUCAUGUUUGCAGUUUCACC*********

rsa-miRn3

ACACAAAGUCAAGCUAGAGACUUAAAACAAGCUCACUUGGGGAGGAAGUCCCAUGUUUAUCCAUUGUAUAUAUUUUUUU
UUAUCUUUCUUUCUUUUAAGUUGUUUAAUAAGUAUGGUUGAGAUUUUCAGGUUGGGUGUA
((((((((((.(((((...(((((((((((.((.....((..((((((...............................
....))))))..))....))))))))..))))).)))))..))))))........)))).
**********AAGCUAGAGACUUAAAACAAG**
****
****
*********************UGUUUAAUAAGUAUGGUUGAG*****************

rsa-miRn4

UUGAUGUAUUAUAUAUGUAUAUGCCUGGCUCCCUGUAUGCCAUACACUAAGUUCAUCGACCAUCGGCGACCUCGGUGGA
UGGCGUAUGAGGAGCCAAGCAUAUUCUCAGCAUAGACAUAUUU
...((((......(((((((((((.(((((((..(((((((((.(((..((.((..((.....))..)).))..))).)
))))))))..))))))).)))))).....))))).))))....
********************AUGCCUGGCUCCCUGUAUGCC**************************************
****
****
GCGUAUGAGGAGCCAAGCAUA******************

rsa-miRn5

GGATUGGUGACGGUGGUGGUGCGAACTGATTGGCTGACACCGCCACGTGAATTTTCACGGTTGGCTTGTGAGCAGGGAT
CGGGCGCCGCCAUGGAAGUCGCCACC

.(((((.((((((.(((.(((((.((((((.(((.(((..(((((((((....)))))..)))).))).)))...)))
))).))))))))..))))))).)))))).

****UGGUGACGGUGGUGGUGCGA***
****

****
***GCGCCGCCAUGGAAGUCGCCAC**

rsa-miRn6

ACGAGAGAGAGACAGGGAACAAGCAGAGCAUGGAUUGAGUUUUACCAAAACAUCAAAUGACUAUGUUUGGUCUCUUCCC
AUGCACUGCCUCUUCCCUGGCUCCCUCUUUC

..(((((.(((.((((((((...((((.((((((.((((((((.....)))).)))).)).(((((....)))))).....))
)))).))))...))))))).))).))))))..

************CAGGGAACAAGCAGAGCAUGG**
****

****
AUGCACUGCCUCUUCCCUGGC**********

rsa-miRn7

UAAAUACUCUAUAUACUGAAGUUUAUACUCUUCAAUCUAGUUAAAAAAUUUCAAACCACAUUGUACAUUUGAAUUUAUG
UACUCUAAACUAUCUUUCAUGUUAUAUAGGAAUUGUAAUAAUUUUUAAUAUUUAUUCUACUAAAGUUUCAUAUAUUGA
CUAGAUUAGUGGAAUUAGGAUCAUAAAAUCUUCAUUAUCUAGAGAAAUGA

......(((((.(((.((((((((((..((((.......(((((((((((.(((.((.....((((((.........)))
))).....(((..........))).....))..)))....)))))))))))....((((((((((((((.........))
))...))))))))))).)))))..))))))..)))))).))).))))))......

**********AUAUACUGAAGUUUAUACUCU**
****
****

****
****
*******************AUCAUAAAAUCUUCAUUAUCUAG********

rsa-miRn8

CAUAUAUACAUAGUACAUAUAUGCCUGGCUCCCUGUAUGCCAUACACUAAGCUCAUCGACCAUCGAGGACUUACGUGGA
UGGCGUAUGAGGAGCCAAGCAUAUUUUCAUAUGCAUAUACAUC

..(((((.((((......((((((.((((((((..(((((((((.((((((((..(.(((.....))))..)))).))).)
))))))))..))))))).))))))).....)))).))))))....

*********************UGCCUGGCUCCCUGUAUGCCA*************************************
****

****
GGCGUAUGAGGAGCCAAGCAUAU***************

rsa-miRn9

ACGAGAGAGAGAGUCGCACUCAUCAUCGGCGUCACGTGAGTTTTACCAAAACATCAAATGACTATGTTTGGTCTCTTCC
C GUACGACGAAGAUGAGCCGACACCCCTCTC

..(((((.(((.((((((((...((((.((((((...(((....((((((.(((..........)))))))))))))...))
)))).))))...))))))).))).))))))..

************GUCGCACUCAUCAUCGGCGUCACG***

****
****
GUACGACGAAGAUGAGCCGACAC********

rsa-miRn10

AGGAAAGUCACUGGAGGCAGCGGUUCAUCGAUCUCUUCCUGUGGAUUUUUUUGUUUGUUUCUUUGUUGUUGUUGGAAAG
AGAUUAAAAGCACGAACAGAUCGAUAAACCUCUGCAUCCAGCGGUUGCCUCUUCAUCAGUC
(((..(..(.(((((.((((.((((.((((((((.(((..(((..((((...((((.(((((..........))))).
)))).)))).)))))).)))))))).)))).)))).))))).)..).)))...........
***********UGGAGGCAGCGGUUCAUCGAUC**
****
****
********************UCGAUAAACCUCUGCAUCCAG********************

rsa-miRn11

UUUUUCCACAGCUUUCUUGAACUUCCUUUUUCAUUUCCCUUCUUUUAAAGAUGUAAUAACUAAAGAUUGUUUUUAAACA
UUUAACACUCUAGAAACAAAAAAAAAAGCUCAAGAAAGCUGUGGGAAAACAUGUCAGGUUCUUUUAAA
((((((((((((((((((.(((..(((((..((((.......(((((....(((.(((........))).)))....
)))))).......))))..)))))..))).))))))))))))))))))).................
UUCCACAGCUUUCUUGAACUU
****
****
***************************GCUCAAGAAAGCUGUGGGAAA********************

rsa-miRn12

AAGGGTTTTCTCTTTTGAGGGAAACUGCCUAAACAAACAUAUCCTCTGGCTCTATCTACTCATGTTGGATCGTCTTCGA
TCTAATCGAATCAAACTTCTATGTTTGAGATCTGATTAGGGTTTTAGCGUAUGCUUUUUAGGCAGUUUCATCCCCCCAA
TTATTGCTCCCTGC
.((((.........(((.((((((((..((((((.((((.(((.(((((((((((...((..(((((((((....))))
)))))....((((((......))))))))...)).)))))))..))).))).)))).)))))))..))))))))).)))...
.....))))....
********************AAACUGCCUAAACAAACAUAUC*************************************
****
****
****
UAUGCUUUUUAGGCAGUUUCA****
****

rsa-miRn13

AAAGCAAGAGUGACAGUUGCUGGAGGCAGCGGUUCAUCGAUCUCUUCCUGAGAAUUUUUUUUGGAAAGAAAGGAAGAU
AAAAAUUAAAAGCAUGAACAGAUCGAUAAACCUCUGCAUCCAGCGGUUUACCUCUUCGUCAAU
...(((((((.(..((..((((((.((((.((((.((((((((((((((.....(((((....)))))...))))))..
.....(((......)))..)))))))).)))).)))).)))))))..))..)))))).))....
******************GCUGGAGGCAGCGGUUCAUCGAU**************************************
****
****
***********************CGAUAAACCUCUGCAUCCAGCG******************

rsa-miRn14

CUGUUUGAUUGGGCUCGGAGAGCUUUAUGGUUUCUCUGGACUGAUUGGCUGACACAACCACUUAGGUGAUUUUGCCUUA

UUGGUUUAUAAGCAAGGAUUGGCUAGAUGACAGUGAGGCUUCUUAGAGCUUCCAAUGGUGAAA

.....(.(((((((((.((.(((.((((((((((.((((((..(((.(((.....(((((...((((......))))..

.)))))....)))...)))..).))))).)))))))))).))).)).))))..))))).)....

******************AGAGCUUUAUGGUUUCUCU**

****

****

**************************AGAUGACAGUGAGGCUUCUUA*****************

rsa-miRn15

AGUCACCGUCGGGCUCGGAUUCGCUUGGUGCAGGUCGGGACUAAUUCGCUGACACAGCCACGUGGCUCUUUUUCUUAUU

GGUUUGUGAGCAGGGAUUGGAUCCCGCCUUGCAUCAACUGAAUCGGAGCUCCCACGGUGAAAA

..(((((((.((((((.((((((.((((((((((.((((((((((((.(((.((((((((...((........))...)

)).)))))..)))))))))).))))).)))))))))).)))))).)))).)).)))))))...

********************UCGCUUGGUGCAGGUCGGGAC**************************************

****

****

**********************CCCGCCUUGCAUCAACUGAAU********************

rsa-miRn16

AGUCACCGUCGGGCUCGGAUUCGCUUGGUGCAGGUCGGGACUAAUUCGCUGACACAGCCACGUGGCUCUUUUUCUUAUU

GGUUUGUGAGCAGGGAUUGGAUCCCGCCUUGCAUCAACUGAAUCGGAGCUCCCACGGUGAAAA

..(((((((.((((((.((((((.((((((((((.((((((((((((.(((.((((((((...((........))...)

)).)))))..)))))))))).))))).)))))))))).)))))).)))).)).)))))))...

**********************GCUUGGUGCAGGUCGGGACUA************************************

****

****

************************CGCCUUGCAUCAACUGAAUCG******************

rsa-miRn17

AUAGAUAUAGAAGGAGAUUCUUUCAGUCCAAUCGUGGAUAGAACAAGAGGGUAGAAAUAUCUGCCGACUCAUCCAUCCA

AACACUCAUGGUUAUGAAACAAGAAAUGUUAAACCACAGUGACUGUGUAUGAAUGAUGCGGGAGAUGUUUUCAUCUCU

CUUUAUCUGUGUUUGGACUGAAGGGAGCUCCUUCUUUAUCUAC

.((((((.((((((((.((((((((((((((.((..((((....(((((((.((((((((((.(((..((((.(((.((

..((((..(((((....((((.....)))).))))).))))....)).))).))))..))).))))))))))...))))

)))))))..)).))))))))))))))).)))))))).))))))).

************GGAGAUUCUUUCAGUCCAAUC**

****

****

****

****

************UUGGACUGAAGGGAGCUCCUU**********

rsa-miRn18

CUGUUUGAUUGGGCUCGGACUCGCUUGAUGCAGGUCGGGACUGAUUGGCUGACACAACCACUUAGGUGAUUUUGCCUUA

UUGGUUUAUAAGCAAGGAUUGGCUCCCGCCUUGCAUCAACUGAAUCAGAGCUUCCAAUGGUGAAA

.....(.(((((((((.((.(((.((((((((((.((((((..(((.(((.....(((((...((((......))))..
.))))).....)))...)))..).))))).))))))))))).))).)).))))..))))).).....
********************UCGCUUGAUGCAGGUCGGGAC**************************************
****
****
************************CCCGCCUUGCAUCAACUGAAU********************

rsa-miRn19

TGTGGTAGAGGATCTCGACAAUGGAUGUAUGAUAUGAUGGAUCCAGUCAACGGCUGUAAUGACGCCACGUCAUUAGAAC
AAATCTTCCTTTCCATUAUUAAUCUGUAUGUUGUUUCGAGCTCTTTCTCTACCGTC
..(((((((((.((((.(((((..(((.((((((((((((..(((((...)))))((((((((...))))))))(((.
.....))).....)))))))))))).)))..))))).))))....))))))))))..
********************AUGGAUGUAUGAUAUGAUGGA**************************************
****
****
****************UAUUAAUCUGUAUGUUGUUUC********************

rsa-miRn20

GACAAGGATTCTTCGACGGGAAGGGGCTTTCTCTTTTGAGGGGAATGTTGTTTGGCTCGAAGACTCTAGCTAGTGAGTT
TTACCAAAACATCAA
.....(((.(((((((....(((.(((.(((((((...))))))).))).)))...))))))).))).....))))).
))).)))...)))).
************TCGACGGGAAGGGGCTTTCTCT***
****
GGAATGTTGTTTGGCTCGAAG**************
****

rsa-miRn21

TTTGGTAGGCGTTGATAGTGUCGGACCAGGCUUCAUUCCCCAGATGATGCGATGCAATTTGGAGCAGCTTTTATGCATC
ATACTCCTTTGTGGAAUGUUGUCUGGCUCGAGGCTCGTTATTTGCCTTAACCC
...((((((((.((((((((.((((((((((((((((((((((.((((.((((((....((.....))....))))))
.)).)).))))))))))))).))))))))))).)))).))))..))))))..))).
********************UCGGACCAGGCUUCAUUCCCC**************************************
****
****
************GGAAUGUUGUCUGGCUCGAGG********************

rsa-miRn22

GGGGGGCUCAGAUAAGCUGCCAGCGUGAUCUUAACUGAUUGGCUGACACAACCACAUAGGUACUUUUGCCUUAUUGGUU
UGUAAGCAUGGAUUUCAGAUCAUGUGGCAGUUUCAGAAUCGGAGCCUCCGAUGGUGAAA
.((((((((.((((((.(((((((((..((((((..(((.(((.(((.(((((...(((((....)))))...))))))
)).)))...)))..).)))))..))))))))).)))))).))))))))..........
*************AAGCUGCCAGCGUGAUCUUAAC**
****
****
**************UCAGAUCAUGUGGCAGUUUCA***********************
